# Quantitative imaging and semiotic phenotyping of mitochondrial network morphology in live human cells

**DOI:** 10.1371/journal.pone.0301372

**Published:** 2024-03-28

**Authors:** Sophie Charrasse, Victor Racine, Charlotte Saint-Omer, Titouan Poquillon, Loïc Lionnard, Marine Ledru, Christophe Gonindard, Sandrine Delaunois, Karima Kissa, Richard E. Frye, Manuela Pastore, Christelle Reynes, Mathilde Frechet, Hanane Chajra, Abdel Aouacheria

**Affiliations:** 1 ISEM, Institut des Sciences de l’Evolution, UMR 5554, Université Montpellier, CNRS, IRD, Montpellier, France; 2 QuantaCell SAS, Institute for Regenerative Medicine and Biotherapy (IRMB), Saint Eloi Hospital, Montpellier University Hospital, Montpellier, France; 3 Clariant Active Ingredients, Toulouse, France; 4 VBIC, INSERM U1047, Université de Montpellier, Montpellier, France; 5 Autism Discovery and Treatment Foundation, Phoenix, AZ, United States America; 6 STATABIO BioCampus, Université de Montpellier, CNRS, INSERM, Montpellier, France; 7 Institut de Génomique Fonctionnelle (IGF), Université de Montpellier, CNRS, INSERM, Montpellier, France; POSTECH - Pohang University of Science and Technology, REPUBLIC OF KOREA

## Abstract

The importance of mitochondria in tissue homeostasis, stress responses and human diseases, combined to their ability to transition between various structural and functional states, makes them excellent organelles for monitoring cell health. There is therefore a need for technologies to accurately analyze and quantify changes in mitochondrial organization in a variety of cells and cellular contexts. Here we present an innovative computerized method that enables accurate, multiscale, fast and cost-effective analysis of mitochondrial shape and network architecture from confocal fluorescence images by providing more than thirty features. In order to facilitate interpretation of the quantitative results, we introduced two innovations: the use of Kiviat-graphs (herein named MitoSpider plots) to present highly multidimensional data and visualization of the various mito-cellular configurations in the form of morphospace diagrams (called MitoSigils). We tested our fully automated image analysis tool on rich datasets gathered from live normal human skin cells cultured under basal conditions or exposed to specific stress including UVB irradiation and pesticide exposure. We demonstrated the ability of our proprietary software (named MitoTouch) to sensitively discriminate between control and stressed dermal fibroblasts, and between normal fibroblasts and other cell types (including cancer tissue-derived fibroblasts and primary keratinocytes), showing that our automated analysis captures subtle differences in morphology. Based on this novel algorithm, we report the identification of a protective natural ingredient that mitigates the deleterious impact of hydrogen peroxide (H2O2) on mitochondrial organization. Hence we conceived a novel wet-plus-dry pipeline combining cell cultures, quantitative imaging and semiotic analysis for exhaustive analysis of mitochondrial morphology in living adherent cells. Our tool has potential for broader applications in other research areas such as cell biology and medicine, high-throughput drug screening as well as predictive and environmental toxicology.

## Introduction

Mitochondria are membrane-enclosed organelles, ubiquitously found in eukaryotes. Originally derived from endosymbiotic bacteria [1], they play a vital role in ATP production as well as in many other cellular processes. These include Ca^2+^ homeostasis, synthesis of various bioactive molecules, reactive oxygen species (ROS) signaling and apoptosis [2–4]. Mitochondrial markers are among the first to vary upon homeostasis disruption, when cells are put under increasing amounts of stress (through toxicant exposure, oxygen deprivation, changes in pH, temperature or nutrient availability) [5–9], after infection [10–12], during disease (often before pathological processes are set in) [13–21] and aging [22–26]. By sensing and responding to changes in the cellular environment, mitochondria initiate and orchestrate adaptive responses that extend well over cellular boundaries to impact tissue, organ and ultimately organism physiology [27, 28].

The ability of mitochondria to signal cellular ‘safety’ or ‘danger’ is related to the fact that they are motile organelles frequently undergoing fission and fusion events [7, 28, 29], two processes collectively referred to as ‘mitochondrial dynamics’. As first noted more than a century ago [30], mitochondria change their morphology according to developmental stage, tissue type and metabolic need. The name of the organelle itself (originally coined in 1898 by Carl Benda using the Greek words for ‘thread’ and ‘grain’) nicely conveys up the idea that mitochondria can be found as solitary dots or in the form of larger networks within cells. Since those early observations, fluorescence microscopy of live cells, labelled with mitochondria-targeted fluorescent proteins or dyes, has largely confirmed the highly dynamic nature of these organelles. Through controlled transport and positioning, mitochondria can be actively recruited to subcellular sites of high energy demand [31, 32]. Moreover, at any given time, the overall structure of the mitochondrial network reflects the net balance of counteracting activities of proteins that mediate either fusion of the outer and inner mitochondrial membranes, or constriction and splitting of the organelle [32, 33]. Mitochondrial fusion produces elongated and interconnected networks, with functional mitochondrial pools maintaining a sufficient supply of ATP [34–36]. In contrast, fission triggers mitochondrial fragmentation so as to pull apart depolarized or dysfunctional mitochondria, which can then be removed by mitophagy [37]. Hence, how healthy a cell is can be judged by the shape of its mitochondria: mitochondria of healthy cells are generally mobile, tubular-shaped and tend to form networks of various topology and complexity, whereas cells undergoing stress or entering apoptosis often display fragmented or swollen mitochondria, marked by concurrent disruption of metabolism, membrane potential, Ca^2+^ signaling and increased ROS levels [38–40]. ROS overproduction alter mitochondria themselves [41], thereby bridging loss of mitochondrial fitness to unhealthy aging [26] and/or impaired cellular resilience [42].

Here, we designed and used MitoTouch, a new tool for analyzing mitochondrial morphology and network connectivity in adherent mammalian cells in culture. We validated this tool and its associated workflow (MITOMATICS) by performing deep analysis of mitochondrial features in normal versus stressed or diseased human skin cells. For the first time in the quantitative imaging field, Kiviat-graphs (spider plots) and a semantically-grounded visual display approach were used to synthesize complex, multivariate data. Since live cell confocal images can be collected from virtually any type of adherent cells of eukaryotic origin before segmentation and automatic processing, we believe that our strategy can find many applications including high-throughput drug testing, predictive and environmental toxicology and cosmetology.

## Material and methods

### Cell culture and treatments

NHDF (C-12302) and NHEK (C-12003) (purchased from PromoCell, Germany) were routinely grown in Fibroblast Growth Medium 2 (Ready-to-use) (C-23020) and Keratinocyte Growth Medium 2 (Ready-to-use) (C-20011) media, respectively. During UVB irradiation and microscopy, NHDF were cultured in phenol-free Fibroblast Basal Medium-2 (C-23225) with supplementation of Growth Medium 2 SupplementMix (C-39325) and NHEK in phenol-free Keratinocyte Basal Medium (C-20216) and Growth Medium 2 SupplementMix (C-39016) (PromoCell, Germany). Skin human melanoma fibroblasts Hs 895. T (CRL-7637) and paired normal fibroblasts Hs 895.Sk (CRL-7636) (purchased from ATCC; LGC Standards) were cultured in Dulbecco’s Modified Eagle Medium (DMEM) or Phenol-free DMEM with Glutamax and 4.5g pyruvate, 10% heat-inactivated Fetal Bovine Serum (HI-FBS) and 1X penicillin-streptomycin solution (Fisher Scientific, Illkirch, France). All cells were cultured at 37°C in a humidified atmosphere with 5% CO_2_. For experiments involving UVB irradiation, cells were placed within a BS-02 UV irradiation chamber equipped with UV-Mat dosimeter (Dr. Gröbel UV-Elektronik GmbH, Ettlingen, Germany). The lamp emits UVB with a peak at 311–312 nm and partially excludes shorter wavelengths, such as UVA. To assess the effects of selected chemicals on mitochondrial morphology and connectivity, cells were cultured in complete medium supplemented with 50 μM Fipronil (46451), 20 μM Imidacloprid (37894), 50 μM H_2_O_2_ during 24h (H1009), 1500 μg/L Glyphosate during 6h (45521) or vehicle alone (DMSO) (Merck, St Quentin Fallavier, France). Human osteosarcoma U2OS cells were treated with vehicle (0,5% DMSO) or 20μM CCCP for 2h 30 min before staining and acquisition. Skin fibroblasts were pretreated with 1500 ppm of Bergaphen (Clariant Active Ingredients, Toulouse, France) 6h before oxidative stress induction by H_2_O_2_ exposure.

### Cell staining

Mitochondrial morphology was examined in NHDF and NHEK cells after 1h of incubation at 37°C with (respectively) 375 or 500nM MitoTracker Red FM (M22425) diluted in phenol-free culture medium in presence of 2μg/mL Hoechst 33342 (H21492). Cells were then washed twice with PBS and labelled with 5 μg/ml CellMask Green Plasma Membrane Stain (C37608) for 5 min at 37°C. U2OS cells were labelled with 250 Mitotracker Deep Red FM (M22426). All fluorescent dyes were purchased from Thermofisher Scientific (Life Technologies SAS, Courtaboeuf, France).

### Live-cell fluorescence microscopy

Live cells were imaged and irradiated on optical quality glass bottom FluoroDish Cell Culture dishes (WPI) containing phenol-free culture media to reduce background fluorescence. The images were acquired using an Andor Revolution Imaging System including a Nikon Ti microscope and the Andor CSU-W1 Spinning Disk Confocal Scanner. Cells were imaged live at 37°C using a 60x oil objective (Nikon CFI PLAN APO LABDA 1.4 NA oil) with magnification set to 1 x zoom (pixel size = 0.108333 μm). Green fluorescence from CellMask Green Plasma Membrane Stain was measured using a 488 nm excitation laser and a single band emission fluorescence filter 520/560. Red fluorescence from MitoTracker Red FM was detected using a 561 nm excitation laser and a single band emission fluorescence filter 607/36. Blue fluorescence from Hoechst 33342 was measured using a 405 nm excitation laser and a single band emission fluorescence filter 447/60. Hardware and 2D fluorescent image acquisition were controlled by Andor IQ3 software. Note that subsequent quantification of mitochondrial shape and network properties was performed on a single optical XY plane (i.e., on a single optical section along the z-axis, a well-suited procedure for analysis of cultured cells having a relatively flat morphology [43–48]). Image acquisition has been optimized to provide high resolution and image quality for accurate analysis. In particular, laser power, detector filtering and exposure time were adjusted to maximize signal without saturation, while also minimizing background signal and photo-bleaching (by imaging the channels in the following order: red, green and blue). Exposure time, gain and laser intensity were identical between samples across an experiment.

### Software implementation

A home-made software solution was developed with Matlab (Mathworks(R)) using the Image processing toolbox and the parallel processing toolbox. A graphical user interface was designed using GUIDE to help users loading their folders to analyze, testing selected images and viewing the results. The output results are made of all the segmentation masks, a.csv file with all the quantitative results of the folder, where each line corresponds to the feature extracted per cell. The threshold of the cell marker can be changed by the user. The MitoTouch software is freely available under the GNU General Public License v3.0 from https://github.com/quantacell/MitoTouch.

### Quantification of mitochondrial parameters with MitoTouch

#### Feature measurements

Descriptors are summarized in Table 1. All mitochondrial features are summed (for areas and lengths) or averaged (for all other features) in order to quantify corresponding cells. Note that calculations were made per experiment, i.e., identical seed culture, same day of image acquisition at the microscope, different conditions like control or stressed, statistics derived from data obtained on pooled microscopic fields for a given condition.

**Table 1:** List of features computed by MitoTouch and their definition.

Feature	Definition
***Cellular Parameters*** ^***a***^	
Area	Total number of pixels occupied by the cell
Mean intensity	Cellular mean intensity on cell channel
Max intensity	Cellular max intensity on cell channel
Perimeter	Perimeter length in pixels
Compaction	MinorAxisDiameter/Majoraxisdiameteroncellellipse
Roundness	sqrt(4*Area/pi)/(Perimeter/4*pi)
***Mitochondrial Cluster Parameters*** ^***b***^	
Fractal2	Fractaldimensionfora4*4pixelssquare
Fractal8	Fractaldimensionfora16*16pixelssquare
Fractal32	Fractaldimensionfora64*64pixelssquare
Count	Number of mitochondria cluster
Area	Mean of mitochondria cluster area
Elongation	MajorAxis/MinorAxisDiameteronmitochondriaclusterellipse
Compaction	MinorAxis/MajorAxisDiameteronmitochondriaclusterellipse
Roundness	Meanofsqrt(4*Area/pi)/(Perimeter/4*pi)
Euler Number	Mean of Euler Number of each mitochondrial cluster
Mito Mean intensity	Mean intensity of mitochondrial cluster
Mito Max intensity	Mean of Max intensity of mitochondrial cluster
Perimeter	Mean perimeter of mitochondrial cluster
Solidity	Perimeter of the convex hull / Perimeter of the object
***Mitochondrial Skeletonized Parameters*** ^***c***^	
Width	Mitochondria width compared to skeleton
Length	Total skeleton length
Branch Points	Number of skeleton branching points
End Points	Number of skeleton ending points
Branch Points Ratio	Branchingpoints/(Branchingpoints+endingpoints)
***Isolated Mitochondria*** ^***d***^	
Compaction	MinorAxis/MajorAxisDiameterofisolatedmitochondria
Elongation	MajorAxis/MinorAxisDiameterofisolatedmitochondria
Roundness	Sqrt(4*Area/pi)/(Perimeter/4*pi)ofisolatedmitochondria
Length	Mitochondrial length
***Mitochondrial Localization*** ^***e***^	
Distance To Membrane	Distance between mitochondria and cell membrane
Distance To Nuclei	Distance between mitochondria and nuclear envelope
Distance Ratio	DistToNuclei/(DistToCellMembrane+DistToNuclei)

^a^These features are extracted from the Cell mask.

^b^These features are extracted from the Mitochondria cluster mask and the red channel.

^c^These features are extracted from the Mitochondria Skeletonized mask and the red channel.

^d^These features are extracted from the Isolated Mitochondria mask and the red channel.

^e^These features are extracted from the Mitochondria Skeletonized mask, Cell mask and the Nuclei mask.

#### Description of the fractal features

The Box-counting method is useful to determine fractal properties of a 2D image. If C denotes the mitochondria mask of a given cell, it counts the number N of 2-dimensional boxes of size R needed to cover the nonzero elements of C. The box sizes are powers of two: R=[2^1,2^2,…2^5], where P is the smallest integer such thatmax(size(C))<=2^P. If the sizes of C over each dimension are smaller than 2^*P*, C is padded with zeros to size 2^*P* over each dimension (e.g., a 320-by-200 image is padded to 512-by-512). The output vectors N and R are of size *P*+1. The gradients of the logarithm of N and R are calculated (gradient(log(N))andgradient(log(R))). The fractal dimensions of the cell are calculated as FD=−gradient(log(N))./gradient(log(R)). In this study, we only used values corresponding to P = 2, 4 and 6, noted Fractal2, Fractal8, Fractal32.

### Segmentation steps

#### Nuclei segmentation

Each multichannel image was processed separately but using parallel processing. Note that the current software version is not configured to process composite of stitched images. The DAPI channel was the first to be extracted from the individual image. This channel was filtered with a bandpass using a gaussian filtering with sigma equal to 3 (to remove sharp objects) and 12 (to remove the background). Resulting channel was thresholded at a level of its median value. Objects with a size lower than 2000 pixels were removed. Holes inside segmentation objects were filled and the obtained mask was dilated with a disk kernel of 3 pixels radius. Touching nuclei were separated using a watershed strategy. Objects on the border were removed. Remaining objects were considered as nuclei.

#### Cell segmentation

A watershed strategy was used to extend segmentation of the nuclei into using the intensity of the green channel. The opposite of the green channel was considered as the map of the watershed, but pixels inside a nucleus were set to -Infinity in order to force nuclei to be the seeds of the cells. Obtained watershed tends to oversegment the cell. First, segmentation areas intersecting nuclei were identified by the label of the nuclei. Then a procedure was developed to aggregate iteratively residual segmentation areas in touch with labeled cells. When a residual segmentation area was in touch with several labeled cells, then it was affected to the cell label showing junctions with the lower channel intensity. Once all pixels of the cell mask were labelled to nuclei, areas where channel intensity was lower than a user-defined value were excluded from cells. Obtained mask was a labeled mask associated with the labelled nuclei mask.

#### Mitochondria cluster segmentation

The channel was filtered with a bandpass using a gaussian filtering with sigma equal to 1 (to remove sharp objects) and 4 (to remove the background). The mask of the mitochondria cluster was obtained as the threshold at 1 standard deviation of the filtered channel. Objects of less than 10 pixels were filtered out. Mitochondria cluster masks were superimposed to the cell mask so that mitochondria can be labelled according to their corresponding cell.

#### Mitochondria skeletonized segmentation

Mitochondrial clusters were separated in order to individualize mitochondria. A skeletonization operation was applied to the Mitochondria clusters. In the skeleton, the ending points and the junction points were separated from the rest of the skeleton pixels. A mitochondria skeleton was defined as the branch between an ending point and a junction point, or 2 junction points or 2 ending points. All mitochondria skeletons were labeled independently in each cell.

#### Isolated mitochondria segmentation

Using a watershed strategy seeded to the mitochondria skeletons, the mitochondria clusters were resegmented such that all pixels of a cluster were labeled to be associated to the mitochondria skeleton. The obtained mask was the isolated mitochondria mask. All pixels of the cluster mask were present in the Isolated mitochondria mask but with a different labelling.

#### Data representation and statistical analysis

Distribution of normalized morphological features obtained from MitoTouch analysis were presented as a MitoSpider plot created in Excel. T-tests were computed using Excel and the MitoSpider plots were annotated by hand to represent the level of significance (P-value) through circles of varying diameter (the larger the circle, the greater the statistical significance). All other statistical and machine learning analyses were performed with the R project version 4.1.2 (2021-11-01). Raw data related to statistics are available as S1 File.

#### Principal component analysis

This unsupervised method summarizes all feature information into novel features (obtained from linear combinations of the original descriptors) that maximize the variance of the data. PCA creates a number of dimensions equal to the number of features and with decreasing importance. The first dimensions allow to visualize similarities and dissimilarities between samples and to interpret those characteristics with respect to original features. The selected features were then tested with the R function *prcomp* [49].

#### Linear discriminant analysis

This supervised method uses, like PCA, linear combinations of all features in order to separate known classes. Novel features are obtained by maximizing the between-class variability (making classes as parted as possible) and minimizing the within-class one (so that classes are as tight as possible) to assign observations to target classes (prediction). The number of new discriminant axis is equal to the number of target classes minus one. The selected features were tested with LDA using the R package MASS [49, 50]. A cross-validation step that separates the observations in two groups (training dataset on which the model is optimized and test dataset on which the model is validated) was performed to get more robust results.

## Results

### Overview of the MITOMATICS workflow

**Fig 1:**
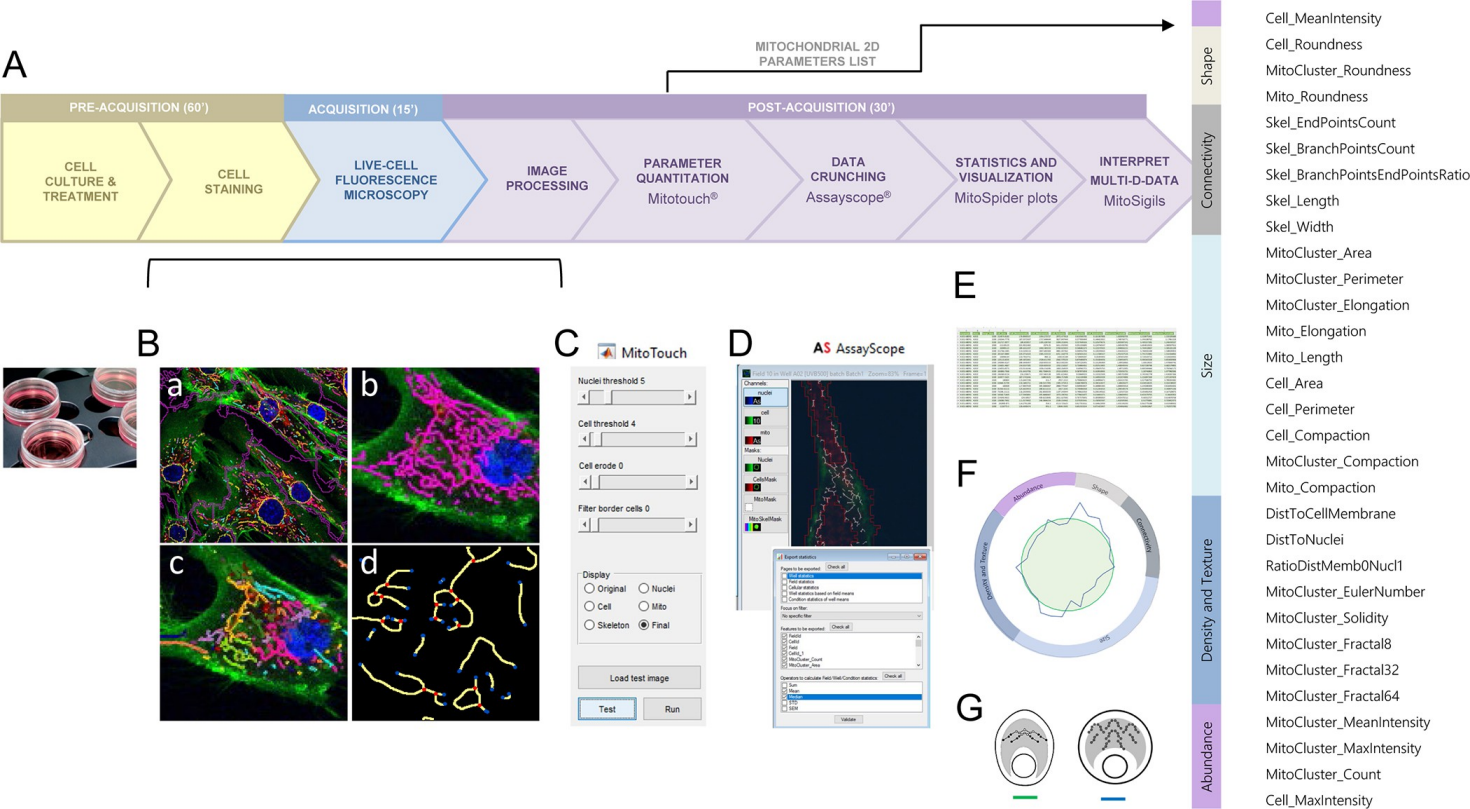
Overview of the MITOMATICS pipeline. **(A)** Main steps of the automated analysis of mitochondrial morphology using the MITOMATICS workflow are presented. Indicative durations needed for the pre-acquisition, acquisition and post-acquisition steps are outlined. See main text for details. **(B)** Fluorescent images of live cultured cells with labeled mitochondria, nucleus and cell contour are captured and loaded into MitoTouch as separate TIFF files. The images are segmented and a total of 31 morphological and texture features (see list on the right) are extracted for each frame. The values can be plotted for a particular cell or for all cells present in a given microscopic field (a), at the level of the whole mitochondrial population (b) or for mitochondrial subnetworks (c). **(C)** MitoTouch offers the option of manually adjusting threshold values until achieving visually appealing segmentation results for nuclei, mitochondria and cell limits. A filter option is also present that can be used to remove cells touching image borders. A skeleton with branch and end points is constructed to represent the spatial structure of mitochondria and their subnetworks (d). Note that MitoTouch processing of a single confocal image lasted on average 10s on a computer with a modern CPU (intel Core i7) and 16 GB RAM. MitoTouch was implemented in MATLAB using custom written scripts. **(D)** The Assayscope software enables to crunch data from MitoTouch output files (i.e., extract data, rename items, generate tables and prepare export files). Statistics and visual exploration of the crunched data. After image processing and data analysis, the multidimensional (multi-D) data are statistically analyzed **(E)** and visually represented **(F)** in the form of spider diagrams (MitoSpider plot) where the reference condition is standardized by a green circle for comparison with a different cellular state (blue line). **(G)** A schematic ‘MitoSigil’ is then associated to the plot to facilitate interpretation (see explanations in S2 and S3 Files).

Objective parameterization of mitochondrial morphology requires sophisticated and robust methods. We developed a pipeline called ‘MITOMATICS’ for fully automated quantification of mitochondriome morphology at the level of single cells. This high-content screening (HCS)-oriented pipeline is based on fluorescence microscopy (with either standard wide-field epifluorescence illumination or confocal microscopes) and computational calculations of a comprehensive set of shape descriptors using a novel software, MitoTouch (Fig 1). Briefly, live-cell 2-dimensional (2D) fluorescence microscopy images are collected from cultured cells stained with three different vital dyes that respectively label mitochondria (in red, using a mitochondrial membrane potential-dependent probe), plasma membranes (in green) and nuclei (in blue) (see pre-acquisition step in Fig 1A). A set of single stack images is generated (acquisition step) before image processing using a custom ImageJ macro (suitable for batch processing) and identification by MitoTouch of the stained objects present in the image (Fig 1B, panel a). MitoTouch visual interface has tunable parameters that enable users to fine-tune image details (Fig 1C). The next step is automatic calculation of 2D descriptors associated with the identified mitochondria (Fig 1B, panel b) or mitochondrial clusters (panel c). Since there is no scientific consensus on the definition of mitochondrial morphology, MitoTouch does not assess mitochondrial phenotypes using simplistic, subjective categories (e.g. ‘punctate’ or ‘fragmented’ mitochondria, ‘tubular’ or ‘intermediate’ mitochondria, ‘networked’ or ‘filamentous’ mitochondria). Rather, the software extracts a total of 31 features associated with geometrical (e.g., size, shape, connectivity) and non-geometrical cues (including texture and intensity) that provide an accurate mathematical characterization of mitochondrial morphology and network organization in relation to cellular parameters (for the exhaustive list of descriptors, see side part of Fig 1). For analysis of mitochondrial network architecture, MitoTouch generates a topological skeleton (Fig 1B, panel d) (in which skeleton points are equidistant from the shape boundary, as shown in the cell depicted in Fig 1D) that is subdivided into branches (or edges), end nodes (blue dots in Fig 1B, panel d) and internal nodes (red dots). Our method also computes the shortest distance of mitochondria to two reference points, namely the nucleus and the cell membrane proximal region. MitoTouch output format is optimized for data navigation and easy manipulation by the Assayscope^®^ software (Fig 1D). This software provides an effective and convenient way to select features and explore the results (data crunching step). Data can also be exported into commercially available software packages (GraphPad, Excel) for statistical analysis (Fig 1E) and advanced graphical representation (spider plot in Fig 1F).

**Fig 2:**
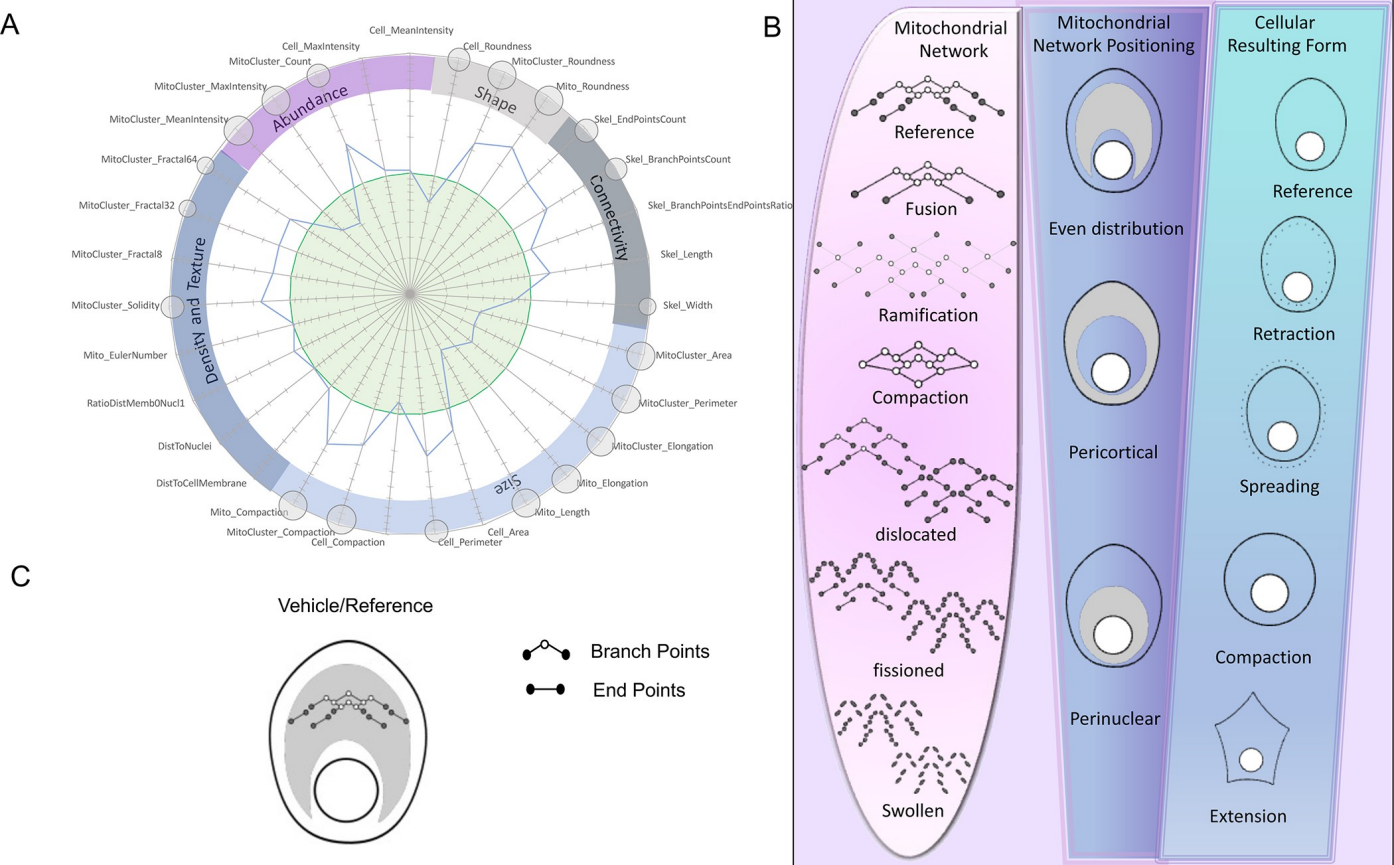
Graphical and schematic interpretations of mitochondrial morphology. **(A)** The 31 parameters measured by the MitoTouch software are reported after normalization on a MitoSpider graph. Parameters values *Vp* (expressed as arbitrary units) are normalized (to give *Vn*) by applying the following formula Vn=(Vp−Vmin)/(Vmax−Vmin). Features are grouped into 5 categories including *Abundance*, *Shape*, *Connectivity*, *Size*, *Density* and *Texture* and compared to a reference (green circle). Significant differences from basal for each condition, n = 3 independent experiments, are notified by small (*: p<0.05), medium (**: p<0.01) or large (***: p<0.001) circles (t-test). **(B)** Schematic representation reflecting the organization of the mitochondria, their positioning in the cell as well as the cellular shape. **(C)** A resulting MitoSigil is then created from this morphological classification (see S2 and S3 Files).

We found that spider plots offered a convenient and intuitive way of presenting the multidimensional (multi-D) data, as most experiments involve comparing a sample dataset to a reference dataset. To our knowledge, despite the interest of spider graphs in synthetizing multivariate data in the form of ‘phenotypic signatures’, this type of visual display has not been used yet in quantitative imaging. We established five ontology classes, i.e., high-level categories that encompass *Shape*, *Connectivity*, *Size*, *Density & Texture* and *Abundance* as defined by the painter Pierre Soulages (1919–1922) in his theoretical conceptualization of shapes [51] (pp. 13–14). Statistical analysis and rational design were used for optimal feature ordering along the circular ‘MitoSpider’ plot showing the Soulages’ dimensions (Figs 1F and 2A). In this representation, each experimental condition is compared to a reference represented by a green circle and normalized to 1 (see methods). The size of external circles represents the level of significance for descriptors displaying statistically significant changes compared to the control condition, with a larger circle indicating more significant P-value than smaller circles (Fig 2A). Last, in order to facilitate the interpretation of the resulting MitoSpider plots in an HCS perspective, we constructed a unique lexicographic code (S1 File) linking all theoretically possible shapes of the plots (S2 File) to diagrammatic representations called ‘MitoSigils’ (Fig 1G and S2 File, panel B). Inspired by George R. McGhee’s work on the universe of shapes [52], this code (termed ‘MitoGrid’) abstractly treats MitoSpider plots (Fig 2A) to build a ‘morphospace’ of mitochondriome morphologies (e.g. fused, ramified, compacted, dislocated, fragmented, swollen) and mean positioning (e.g. pericortical, perinuclear) in a given cellular context (e.g. in a retracted, spread, compacted or extended cell) (Fig 2B). Each conceptual diagram of the morphospace (i.e., each MitoSigil) corresponds to a unique MitoSpider plot configuration and represents a particular state in the spectrum of cellular and mitochondrial shapes (Fig 2C).

### Increasing doses of UVB irradiation alters mitochondrial morphology in live skin cells

**Fig 3:**
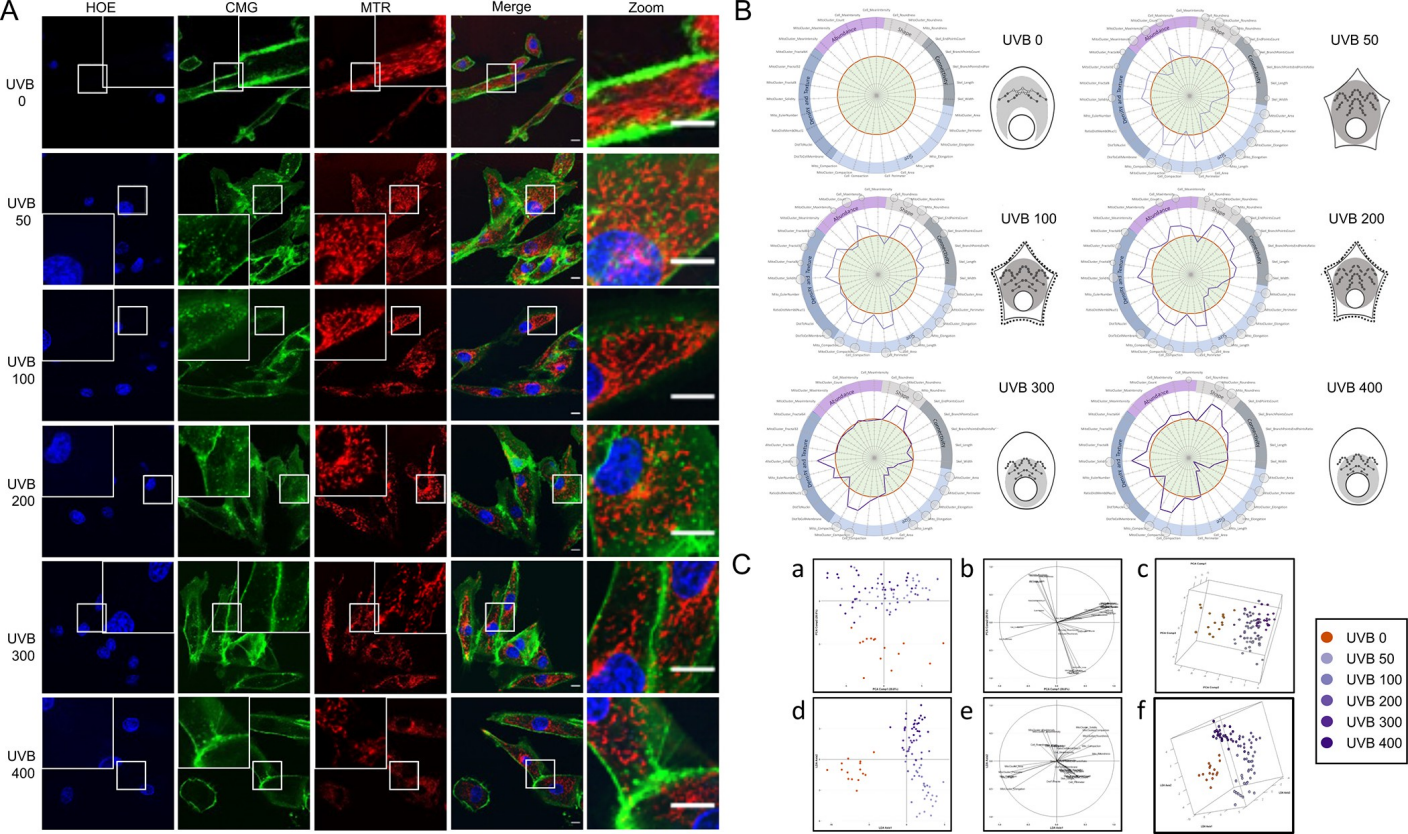
Increasing doses of UVB irradiation disturb mitochondrial morphology in NHDF cells. **(A)** Fluorescent representative imaging of nuclei using Hoechst staining (blue; HOE), membranes with CellMask dye (green; CMG) and cellular distribution of the mitochondria-specific dye MitoTracker Red FM (red; MTR) after irradiation with increasing doses of UVB (50 to 400 mJ/cm^2^). Pictures were taken using live-cell confocal microscopy 6h after irradiation. Single, overlapping images and zoom inserts are presented. Scale bar = 10μm. **(B)** After analysis of a minimum of 300 cells with MitoTouch software, the distribution of normalized morphological features *(Vn)* is represented by a MitoSpider plot. Significant differences with respect to the control condition, n = 3 independent experiments, are notified by small (*: p<0.05), medium (**: p<0.01) or large (***: p<0.001) circles (t-test). Fibroblasts are sensitive to increasing UVB irradiation as shown by the purple broken lines in relation to the reference red circle (no UVB irradiation). Profiles are similar until 200 mJ/cm^2^, and slightly modified with 300 and 400 mJ/cm^2^ as reported by the MitoSpider plots and the associated MitoSigils. **(C)** Projection of the samples onto the 2D space generated by the first two Principal Components (a). PCA2 segregates irradiated samples from untreated ones, independently of the irradiation dose. Correlation of each feature to the first two Principal Components (b). Projection of the samples onto the 3D space generated by the first three Principal Components (c). Projection of the samples onto the 2D space generated by the first two LDA discriminant axis (d). LDA1 splits irradiated samples from untreated ones, while LDA2 highlights the irradiation dose gradient. Correlation of each feature to the first two LDA discriminant axis (e). Note that features parallel to LDA1 have an explanatory role in the observed segregation between treated and untreated samples, whereas those parallel to LDA2 in the categorization of irradiated samples depending on their UVB dose. Projection of the samples onto the 3D space generated by the first three LDA discriminant axis (f). UVB doses are shown in the inset.

We used the MITOMATICS pipeline to characterize the mitochondrial phenotypes of primary normal human dermal fibroblasts (NHDF), which are relatively flat cells. Single-plane cell areas are therefore expected to provide a fair estimation of the number and organization of mitochondria [47, 53, 54]. To test the ability of our integrated pipeline to detect changes in mitochondrial morphology, fibroblasts were irradiated with increasing doses of UVB (50 to 400 mJ/cm^2^) or left untreated. Massive UVB irradiation results in increased ROS levels, which cause oxidative damage to cellular components including mitochondria [41, 55]. By 6h after irradiation, confocal microscopy observation revealed obvious differences in mitochondrial organization compared to the control condition (Fig 3A). Untreated cells show a mitochondrial phenotype with typically long, moderately branched mitochondria, together with smaller clusters of connected mitochondria. In contrast, in irradiated cells the proportion of long mitochondria appears to be greatly reduced, with an overall increase in the number of small mitochondrial clusters and dots, which is indicative of mitochondrial fragmentation. Quantitative analysis using MitoTouch confirmed this shift in all irradiated groups, with mitochondria being rounder and shorter in irradiated fibroblasts (as witnessed by significantly higher compaction and roundness, and lesser length, elongation, perimeter and area) than in control fibroblasts (Fig 3B). Most of these features belong to the *Shape* and *Size* categories, and their modification reflects the same basic effect: post-irradiation mitochondrial fragmentation, as observed in the micrographs (Fig 3A). In addition, our method of analysis with MitoTouch allows to specify when the mitochondria are partially or completely fissioned according to the UV doses (with an increase of skeletal end and branch points), or swollen for the 200 mJ/cm^2^ dose (attested by a significant increase in Skel_Width). This result can be explained by the fact that UVB irradiation tends to deplete the mitochondrial pool from long solitary mitochondria while at the same time increases the proportion of small clustered mitochondria (Fig 3A). For the lowest doses of UVB (< 300mJ/cm^2^), about two-third of the features exhibit positive or negative correlations to UVB dosage, with MitoCluster_Count, MitoCluster_Fractal32, Skel_EndPointsCount, Skel_BranchPointsCount being significantly increased, as well as the Cell_Perimeter parameter, in line with the star-like cellular shape visible on the microphotographs (highlighted by the cognate MitoSigils in Fig 3B). Fibroblasts damaged by UVB irradiation tend to lose their typical elongated shape, which is supported by a statistically significant increase in Cell_compaction (for all UV doses) and Cell_Area (for UVB100 and 200) (Fig 3B). As the UV dose increases (> 300mJ/cm^2^), the fissioned mitochondrial pool tends to move closer to the nucleus as attested by the significant decrease in the mean mitochondrial distance to the nucleus (also rendered by the compact light grey zone in the MitoSigil representations (Fig 3B)). Last, cell-permeable MTR probe accumulates in mitochondrial membranes and quantification of fluorescence intensities suggests that irradiation with 50 and 100 mJ/cm^2^ UVB led to a decrease in mitochondrial membrane potential (MMP), a process commonly associated with lower mitochondrial content, decreased respiration and stress [56].

To take into consideration all the features and samples globally, we performed multivariate analysis, starting with Principal Component Analysis (PCA). As shown in Fig 3C (panel a), the irradiated samples were nicely separated from untreated controls by PCA2. While PCA failed to separate the samples depending on the irradiation strength (Fig 3C, panel c), linear discriminant analysis (LDA) successfully distinguished irradiated samples from untreated ones and irradiated samples according to their UVB dose (Fig 3C, panels d and f). Panels b and b give information about the features that most correlate to the PCA2 and LDA2 axis, respectively (i.e., those that are most likely to be involved in the observed segregation). Overall, our data indicate that UVB irradiation triggers measurable changes in the morphology of the mitochondriome of skin NHDFs. Interestingly, our methodology was able to capture non-monotonic dose-effect relationships, as evidenced by the subtle differences that can be observed between the various MitoSpider plots and MitoSigils according to the UVB dose. LDA projection of the first three discriminant axis onto a 3D graph (panel f of Fig 3C) additionally highlights the non-linear behavior of the dose treatment progression.

### Exposure to pesticides differentially perturbs mitochondrial morphology in live skin cells

**Fig 4:**
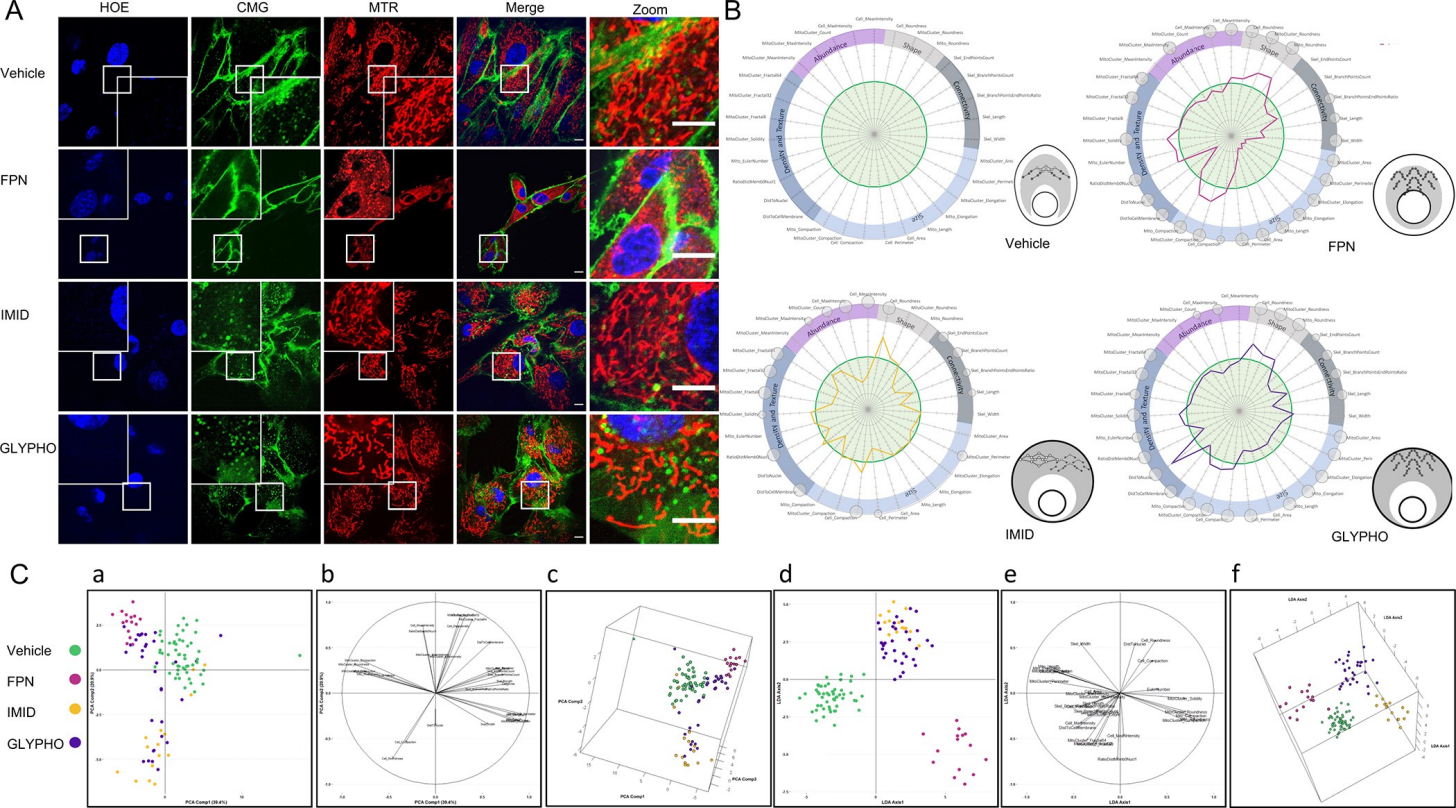
Distinct changes in mitochondrial morphology of NHDF cells after exposure to three different pesticides. **(A)** NHDF cells were treated with vehicle (DMSO), Fipronil, Imidaclopril, or Glyphosate for 6h and stained with HOE (blue), CMG dye (green) and MTR (red) before imaging. Scale bar = 10μm. **(B)** Mean mitochondrial and cellular features measured in cells exposed to FPN (pink broken line), Imidaclopril (orange broken line) or Glyphosate (purple broken line) and compared to the reference condition (DMSO, green line) are reported onto MitoSpider plots. Significant differences from basal for each condition, n = 3 independent experiments, are notified by small (*: p<0.05), medium (**: p<0.01) or large (***: p<0.001) circles (t-test). The resulting ‘mito-signatures’ (MitoSpider plots) and their associated phenotypic representation (MitoSigils) are depicted. **(C)** Bidimensional representation of the samples into the new subspace generated by the first two principal components (a). PCA Comp1 globally separates treated samples from vehicle alone. Different compounds affect differently samples coordinates shifting them away from vehicle more or less markedly and in diverse directions. Correlation of each feature to the first two principal components (b). Projection of the samples onto the 3D space generated by the first three Principal Components (c).). Projection of the samples onto the 2D space generated by the first two LDA discriminant axis differentiates treated samples from untreated controls (d). LDA1 completely splits treated samples from untreated controls. In addition, LDA2 reveals that toxicants acting with different biological mechanisms were differentially clustered. IMID and GLYPHO partially overlap, whereas FPN appears to lie far apart from the other groups. Correlation of each feature to the first two LDA discriminant axis (e). Projection of the samples onto the 3D space generated by the three LDA discriminant axis (f). LDA3 allows to completely separate IMID from GLYPHO, two groups of samples that partially overlapped with each other in the 2D representation. The various conditions are shown in the inset.

Exposure to multiple environmental pollutants (air, water and soil) throughout life has a significant impact on health [57–59]. To test the ability of our methodology to detect changes in mitochondrial morphology induced by cellular stressors other than UVB, cells were treated with three chemical compounds with relevance to environmental toxicology: two insecticides, fipronil (FPN) [60] and imidacloprid (IMID) [61] as well as glyphosate (GLYPHO) [62], the active ingredient of the widely used herbicide Roundup^®^. These three systemic pesticides are easily absorbed by plants or animals and, in these latter organisms, circulate through the blood vascular system and within lymph vessels. Since the noxious effects of these chemicals could be observed not only after ingestion or inhalation but also after skin application, NHDF cells were used for their testing with MITOMATICS. Live-cell imaging of mitochondria show disrupted mitochondrial networks in cells following single exposure to one of these pesticides compared to control cells, with clearly discernable mitochondrial fragmentation induced by FPN (Fig 4A). At the tested concentrations, FPN significantly impacts 29 parameters out of the 31 parameters assessed by MitoTouch, IMID a total ratio of 19/31 and GLYPHO 26/31 (Fig 4B). All the treatments resulted in smaller and rounder cells, as revealed by a statistically significant increase in Cell_Compaction and Cell_Roundness and concomitant decrease in Cell_Perimeter and Cell_Area. For both FPN and GLYPHO, isolated and clustered mitochondria also appear to be rounder, which is indicative of mitochondrial fragmentation. Mitochondrial *Size* features (including MitoCluster_Area, MitoCluster_Perimeter, MitoCluster_Elongation as well as Mito_Elongation and Mito_Area) are dramatically decreased after FPN treatment and, albeit to a lesser extent, after exposure to GLYPHO. Connectivity parameters that account for the branching and extent of the mitochondrial network were also profoundly compromised after pesticide treatment with a greater effect observed with FPN and GLYPHO. MitoSigils of these two pesticides look quite similar, except at the subcellular level where the dislocated pool of fragmented mitochondria tends to be enriched near the cell nucleus after FPN treatment and near the cell membrane after GLYPHO exposure. In contrast, the mito-signature obtained after IMID treatment, as well as its inferred MitoSigil, suggest that treatment with this pesticide triggers partial dislocation of the mitochondrial network, as shown by the reduction in skeleton length and in the number of skeletal branchpoints in association with increased solidity and decreased perimeter of the mitochondrial clusters.

PCA confirmed that cells undergo global mito-cellular modifications upon toxicant exposure (Fig 4C, panels a-c). Samples treated with FPN were tightly grouped, with their positioning explained by variations in MitoCluster_Perimeter, MitoCluster_Elongation, Cell_Compaction Cell_Roundness and RatioDistMemb0Nucl1, among other features. Exposure to IMD dragged the cellular response in a direction opposite to that of FND, mainly due to modifications in Cell_Compaction, Cell_Roundness and Fractal. Samples treated with GLYPHO show more heterogeneous behavior, some of them clustering with untreated samples while others fall closer to FPN or IMD. LDA completely discriminated different toxicant effects and toxicant-treated groups versus control (Fig 4C, panels d-f). IMID and GLYPHO present similarities and partially overlap (panel d) but are well split apart by LDA3 (panel f). When 2-fold cross validation was applied to minimize overfitting, the accuracy of class assignment was still 90%, suggesting a very robust prediction. All in all, MitoTouch is able to measure subtle changes in cellular and mitochondrial shapes induced by single exposure to various pesticides, offering the possibility to discriminate between the mitochondrial effects of chemicals exhibiting mitotoxic properties.

### Identification of a natural ingredient attenuating oxidative stress-induced mitochondrial fragmentation in live human skin fibroblasts

**Fig 5:**
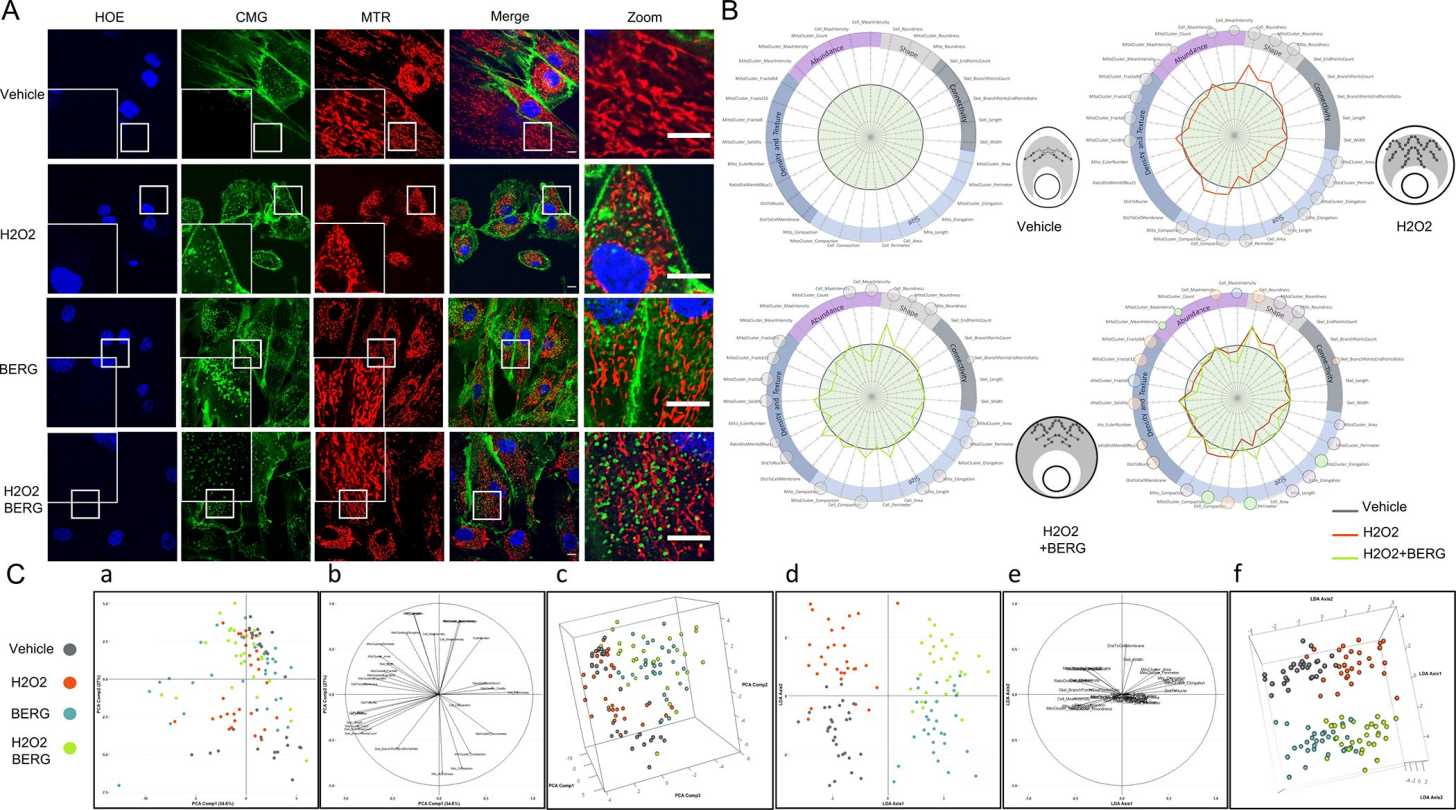
Identification of a natural compound with mitochondrio-protective activity against hydrogen peroxide-induced mitochondrial damage. **(A)** Mitochondrial network of control NHDFs (vehicle), treated with H_2_O_2_ (50μM), Bergaphen (1500ppm), with or without (medium) pre-incubation with the asset of interest**. (B)** Morpho-phenotypic signatures obtained after analysis by MitoTouch, statistical tests, graphs and inferred MitoSigils. Significant differences from basal (grey circle) computed for stressed samples (H_2_O_2_, broken red line) or for samples pretreated with Bergaphen before H_2_O_2_ addition (BERG, broken green line) are notified by small (*: p<0.05), medium (**: p<0.01) or large (***: p<0.001) circles (t-test). Green: restored parameters; violet: partially restored parameters; blue: parameters getting worse in the pretreated group; orange: no protective effect and brown: damaged. The mitochondrial network appears to split in stressed cells (H_2_O_2_; red line) while preconditioning with the natural ingredient (BERG; green line) has protective effects on a number of morphological parameters of the cellular mitochondriomes (bringing the orange line closer to the grey line). The results are representative of three independent experiments, each involving around 500 cells. **(C)** Projection of the samples onto the 2D space generated by the first two principal components. PCA does not allow to separate the four groups (a). Correlation of each feature to the first two principal components (b). Projection of the samples onto the 3D space generated by the first three principal components (c). This view allows to globally differentiate samples treated or not by BERG. Projection of the samples onto the 2D space generated by the first two LDA axis (d). The four groups are successfully discriminated by LDA: LDA1 separates samples that were treated or not by BERG whereas LDA2 distinguishes H_2_O_2_-treated samples versus control samples. Correlation of each feature to the first two LDA discriminant axis (e). Projection of the samples onto the 3D space generated by the three LDA discriminant axis (f). This view confirms the separation of the four groups viewed in the 2D graph.

Acute exposure of NHDFs during 24h to a low concentration (50μM) of hydrogen peroxide (H_2_0_2_), a reactive oxygen species (ROS) donor, induced massive mitochondrial fragmentation (Fig 5A). Quantification using MitoTouch confirmed this result: a total of 20 parameters out of 31 (64,5%) were significantly impacted after H_2_O_2_ treatment (broken orange line in the upper right MitoSpider plot in Fig 5B), with significantly increased Mito(cluster)_Roundness and Mito(cluster)_Compaction, decreased Mito-Length, Mitocluster-Perimeter, Mitocluster_Area and mitochondrial network fractality, compared to the control condition (vehicle alone). MitoTouch analysis revealed that, in addition to undergoing mitochondrial fragmentation, treated cells also become rounder and more compact (as indicated by reduced Cell_Perimeter and enhanced Cell_Roundness).

Next, we investigated whether an extract isolated from *Citrus bergamia* (bergamot) can reduce the deleterious effects of H_2_0_2_ on the mitochondrial network of NHDFs. Cells were preincubated for 24h in presence of Bergaphen-15 before H_2_0_2_ or vehicle treatment for an additional 24h. After Bergaphen-15-preconditioning (green line of the MitoSpider plots depicted in Fig 5B), half of the parameters (12 out of 24) affected by H_2_0_2_ treatment have values either comparable (green circles) or at least significantly closer (violet circles) to control parameter values (Fig 5B, bottom right). Treatment with Bergaphen-15 significantly attenuated H_2_0_2_-induced mitochondrial fission, as indicated by the Mitocluster_Elongation and Mitocluster_compaction parameters that did not differ from the control. Other parameters (e.g. mitochondrial mean length and roundness) were significantly improved in the extract-added, H_2_0_2_-treated condition. Although PCA did not show a clear partitioning pattern of the different treatments (Fig 5C, panels a, b and c), LDA was able to distinguish the four conditions (panels d and f in Fig 5C). In agreement with the MitoSpider plots, the Bergaphen-15-preconditioning influenced features correlated to the first discriminant axis such as Mitocluster_Elongation and Mitocluster_compaction (panels a and b in Fig 5C). Compared to the univariate analysis, LDA was able to highlight variations following Bergaphen-15-preconditioning that were only slightly evidenced by MitoTouch analysis. These results demonstrate that hydrogen peroxide causes massive mitochondrial fragmentation in cultured human dermal fibroblasts and that this damaging effect appears significantly reduced by addition of a bergamot extract.

### Automated discrimination of distinct cell types based on morphological features

**Fig 6:**
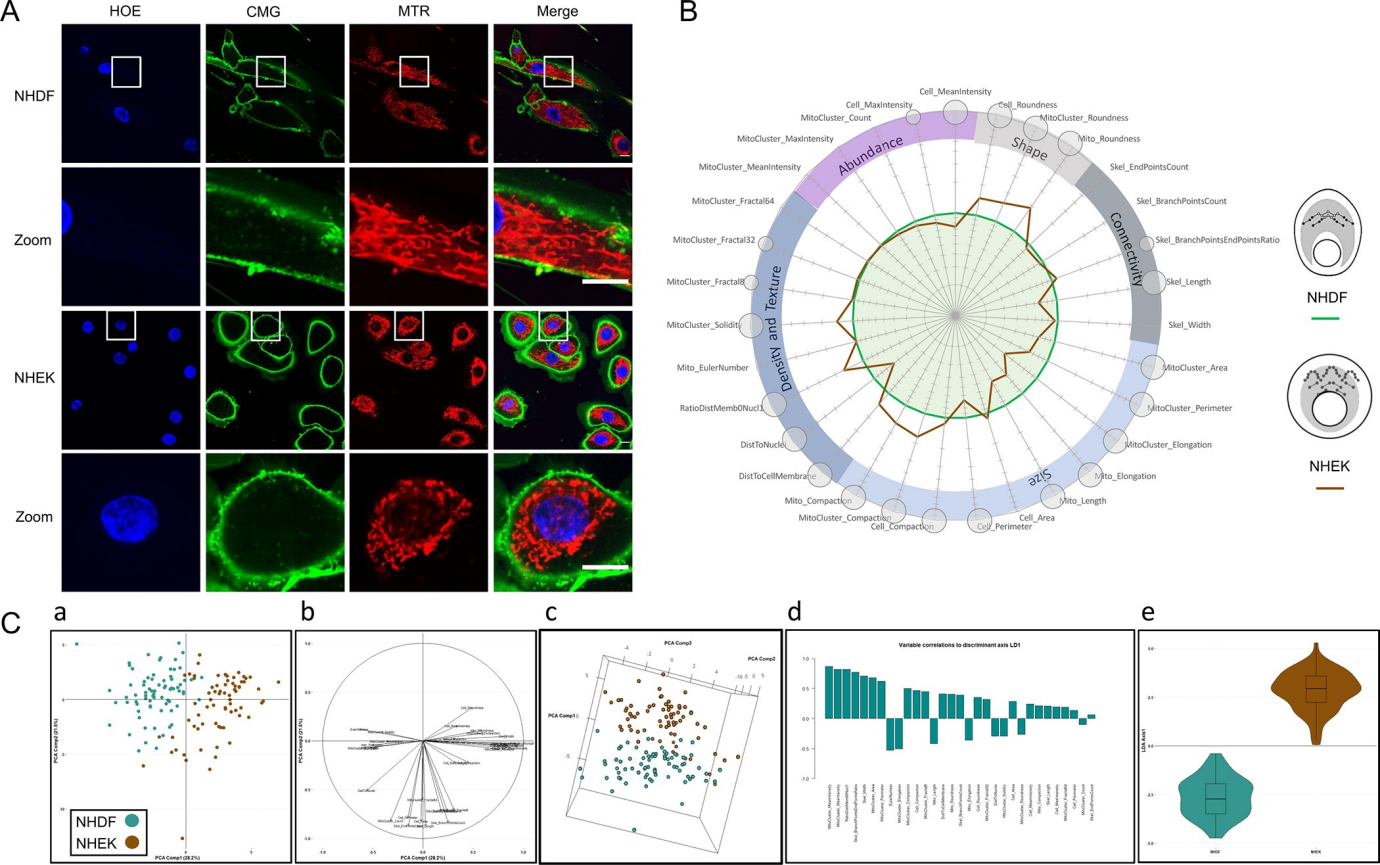
Cultured human fibroblasts (NHDF) and keratinocytes (NHEK) show distinct morpho-phenotypic mitochondrial profiles. **(A)** Representative immunofluorescence images are shown for nuclear (blue), cellular (green) and mitochondrial (red) staining in fibroblasts (NHDF) and keratinocytes (NHEK). Merge corresponds to the superposition of the 3 colored images. The bottom inserts (zoom) correspond to enlargements of the areas surrounded at the top. Scale bar = 10μm. **(B)** MitoSpider plots showing variations in the cellular and mitochondrial morphology in fibroblasts taken as reference (green line) versus keratinocytes (brown line). Significant differences between both cell types are indicated by small (*: p<0.05), medium (**: p<0.01) or large (***: p<0.001) circles (t-test). The inferred MitoSigil reflects the fact that keratinocytes are rounder cells with a less compact and intertwined mitochondrial network compared to fibroblasts. The results are representative of at least 3 independent experiments, each involving around 500 cells. **(C)** Projection of the samples onto the 2D space generated by the first two principal components (a). The two types of cells can be separated by a diagonal line. Correlation of each feature to the first two principal components (b). Projection of the samples onto the 3D space generated by the first three principal components (c). This view confirms the separation of the two groups visible in the 2D graph. Correlation of each feature to the LDA discriminant axis (d). Violin plots showing that both types of cell morphology are well-discriminated (e).

**Fig 7:**
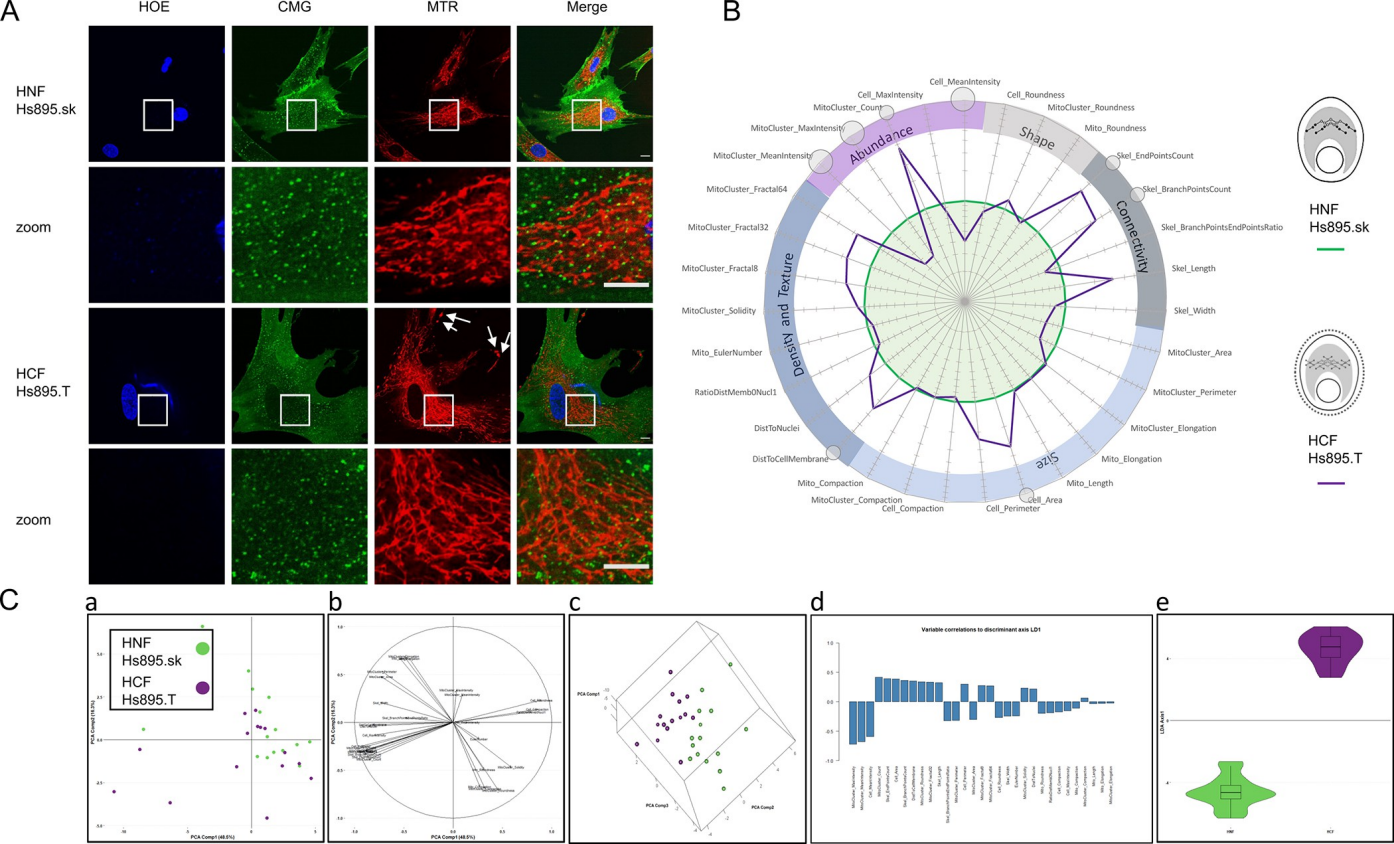
Variations in the mitochondrial network of melanoma-associated and normal skin fibroblasts. **(A)** Human normal fibroblasts (HNF) (Hs895.sk #CRL-7636) and human cancer fibroblasts (HCF) (Hs895.T #CRL-7637) derived from the same patient were stained for nuclei (HOE blue), cellular membranes (CMG) and mitochondria (MTR). White squares indicate zoomed areas shown in insets. Scale bar = 10μm. **(B)** MitoSpider plot and cognate MitoSigil inferred after analysis by MitoTouch show cellular retraction (significant decrease in Cell_Area, small circle *: p<0.05) with concomitant ramification of the mitochondrial network (significant increase in the Skel_EndPointsCount parameter, small circle *: p<0.05) in cancer cells (purple line) compared to their normal counterparts (green circle). The results are representative of at least 3 independent experiments, each involving around 500 cells. **(C)** Projection of the samples onto the 2D space generated by the first two principal components (a). Using PCA, the two types of cells cannot be separated. Correlation of each feature to the first two principal components (b). Projection of the samples onto the 3D space generated by the first three principal components (c). This view allows bona fide separation of both groups. Correlation of each feature to the LDA discriminant axis (d). Violin plots showing that both types of cell morphology are well discriminated (e).

We sought to determine whether differences in overall cellular shape and mitochondriome morphology could be captured by our algorithm. Feature quantification was performed in a pairwise manner using four cell types: normal human dermal fibroblasts (NHDF) versus normal human dermal keratinocytes (NHDK, the other major skin cell type beyond fibroblasts, Fig 6), and a tumor fibroblast cell line (Hs895.T) derived from a patient who had metastatic melanoma matched to an immortalized cell line (Hs895.sk) established from the adjacent normal skin tissue of the same patient (Fig 7). Compared to fibroblasts (green line, taken here as a reference), keratinocytes exhibit a mitochondrial phenotype characterized by shorter and rounder mitochondria (as indicated for instance by the higher values obtained for the Mito_Roundness and Mito_Compaction parameters). Interestingly, our analysis reveals that keratinocytes have a higher density of mitochondria in perinuclear regions in comparison with cell periphery (with a decreased mean distance to the cell nucleus and a concomitant increase in the distance to the cell membrane). The observed gain in solidity may also be due to this perinuclear aggregation of mitochondrial clusters, forming a ‘bulk’ with less defined borders than solitary and well-separated mitochondria. Due to the higher density of mitochondria around the nucleus, their space-filling complexity is also expected to increase, which is corroborated by the correlative increase in fractality (Fig 6B). Morphological analysis of keratinocytes and fibroblasts by MitoTouch generated dissimilar values for cellular size and shape features, with significantly higher roundness and compaction and a lower average perimeter for keratinocytes. This result fits well with the observable multi-sided, circular polygonal shape of keratinocytes and the more elongated aspect of fibroblasts (Fig 6A).

PCA analysis was also able to separate NHDF and NHDK populations (panels a and c of Fig 6C). Descriptors involved in this discrimination can be grouped into three distinct clusters (panel b of Fig 6C): two parallel to the first PCA component and the third having more distinct features and being mainly parallel to the 2^nd^ PCA component. Since there are only two target classes, samples could only be plotted unidimensionally (panel e of Fig 6C). This analysis confirmed absolute separation of the two cell lines. The features most correlated to this discriminant axis were, in addition to those related to the intensity, RatioDistMemb0Nucl1, Skel_BranchPointsEndPointsRatio and Skel_width (panel d of Fig 6C).

When fibroblastic cell lines derived from either tumoral (Hs895.T) or surrounding normal tissue (Hs895.sk) were compared, there were noticeable differences in cellular and mitochondrial morphologies, with the tumor cells displaying an enlarged cellular perimeter, extended mitochondrial network (Fig 7A) and instances of mitochondrial accumulation at membrane protrusions (arrows in Fig 7A), which fits well with their migratory and invasive status [63, 64]. Quantification with MitoTouch confirms these observations with significant increases in cell surface (Fig 7B), number of clustered mitochondria (MitoCluster_Count) and skeletal ends (Ske-EndPointsCount). As in the case of NHDF and NHDK populations, following LDA analysis (panels d and e of Fig 7C) samples could only be plotted on one-dimension violin plots. This analysis confirmed perfect separation of the two cell lines. Original descriptors most correlated to this discriminant axis were, in addition to those related to the intensity, MitoCluster_Count, Skel_EndPointsCount, Cell_area, Skel_BranchPointsCount. Overall, our data show that our mitochondrial analysis pipeline can be applied to establish typical mitochondrial signatures for healthy and diseased cells, in addition to differentially treated cells.

## Discussion

Mitochondria are dynamic and hypersensitive organelles that adapt their number, shape, position, connectivity and movement in response to various stimuli internal or external to the cell [2, 32]. Increased cellular energy requirements have been associated to fusion of mitochondria and extension of their network by branching and reticulation, while in response to stress or cellular damage (for instance after exposure to toxicants [65, 66]), mitochondria were repeatedly observed to fragment into smaller units before being eliminated [67]. Alterations in the morphology or dynamics of mitochondria were also observed during aging [68] and in many human pathologies [3, 13–15, 69] including neurodegenerative diseases [16], amyotrophic lateral sclerosis [17], diabetes [18] and cancer [19–21]. This massive implication of mitochondrial remodeling in pathological states is paralleled by the continuous discovery of pleiotropic roles for mitochondria in biological events as diverse as pollen development [70], animal behavior [71], cell fate [72], neuroplasticity [73], immunity [12] (in connection with the mind [74]) and even light processing through an optical lens-like function [75]. Analysis of the morphology of mitochondria can therefore be considered as a proxy for their function in diverse situations and tissues [29, 76], in a way complementary to measurements of cellular respiration or ROS production. However, the mitochondriome (the mitochondrial content of any given cell) takes the form of a complex landscape exhibiting characteristics such as shape and texture heterogeneity, scale dependence, and multi-parametric definitions of cell morphology that make determination of the mitochondrial shape phenotype notoriously difficult to determine. In this paper, we described a methodology (MITOMATICS) for fast, robust and unbiased analysis of 2D-shape changes in the mitochondrial network of cultured adherent cells. Thanks to a novel algorithm (MitoTouch) that computes multiple quantitative metrics at the mitochondrial and cellular levels, morphospatial data can be extracted from fluorescence images and processed to create innovative, convenient and intuitive multi-D representations (MitoSpider plots) and schematic visualizations (MitoSigils) of cellular mitochondriomes. Our wet-plus-dry pipeline presents a number of advantages over most existing tools, including the following: (i) the use of vital dyes staining virtually all cell types (instead of immunodetection of mitochondrial proteins in fixed cells or fluorescence microscopy of tagged forms after transfection or infection of cultured cells, which may lead to artefacts in the pattern of localization); (ii) automatic object (cell, mitochondrion) segmentation that does not rely on manual adjustment of object delineation, allowing for rapid image processing, analysis and data access (with a time saving factor estimated to 45 compared to a manual method in a model of mitochondrial fragmentation induced by the well-known mitochondrial stressor CCCP [77], see S4 File); (iii) a rich set of morphological descriptors (n = 31, whereas other free image analysis software has limited measurement features, usually less than 10, see S4 File); (iv) a visualization procedure particularly well suited for experiments involving large image data sets and statistically comparing samples of different conditions to a control condition (with a unique ‘MitoGrid’ featuring various mito-cellular configurations). In that respect, it is interesting to notice that the ‘mito-signatures’ obtained so far appear to segregate into discrete families, suggesting that the effects of damaging conditions or noxious treatments might be classified within a ‘morphospace’ of mitochondriome morphologies. More features, calculated in various biological models, will be required to firmly build a quantitative relation between the multiple representations in a reciprocal ontological model and cellular changes in mitochondrial morphology and connectivity. Future improvements can also be expected through culture in 96-well microplates combined to automated pipetting and microscopy to support high-throughput / high-content screening methods, allowing large amounts of imaging data to be produced on different cellular models, while reducing inter-observer variability and interaction time. Note that, for now, our method of automated quantification of mitochondrial morphology appears to be well-suited for 2D cell culture studies, i.e., for relatively ‘flat’ cells where mitochondria appear to be confined to a limited number of planes [43–48]. For thicker cells, it will be necessary to acquire multiple confocal images along their z-axis (3D image acquisition) before projection of the individual z-stack sections into one pseudo-3D-image or, ideally, 3D volume representation of the mitochondria from the z-stack. Moving to 3D cell culture may be envisioned using for instance collagen gels or Matrigel for 3D skin reconstruction or to study cancer cell growth and invasion. Last, live cell image data analysis would be greatly upgraded by designing an all-in-one database-graphical environment including standard and custom statistical analysis (t-tests, PCA, LDA, violin distributions) and information-rich representations using a flexible proprietary interface for visualization of the results. All these enhancements are currently under development.

Due to its strategic location at the interface between the outside world and the internal milieu, the skin shares with mitochondria the ability to sense changes in the environment [78]. This organ (the largest in the human body) is continuously exposed to numerous external biological and environmental factors (e.g. UV radiation, pollutants and microbial insults) and, like mitochondria, it computes and translates the received inputs into physicochemical and biological signals that regulate local and global homeostasis. Skin mitochondria play important roles in health maintenance for instance through melatonin biosynthesis, which in turn influences mitochondrial bioenergetics [79]. In primary human fibroblasts, circadian oscillations in ATP production were found to be tightly coupled to a rhythmic fragmentation of mitochondrial networks [80], whereas in primary human keratinocytes, mitochondrial fragmentation has been associated with aging [81]. Cutaneous cells and their mitochondrial populations are particularly affected by external factors such as UV exposure [41, 55] and toxicants [82] that cause detrimental effects on skin structure and function. Our data establish MITOMATICS as an efficient technology to screen bioactive ingredients that could minimize environment-induced mitochondrial damage in skin cells (for adaptive, anti-pollution skin care). In a recent study, a preliminary version of MitoTouch found that fibroblasts from children with Autism Spectrum Disorder (ASD) with electron transport chain uncoupling rates demonstrated distinct mitochondrial morphology as compared to those with more typical respiratory rates [69]. Furthermore, mitochondrial morphological parameters significantly correlated with the uncoupling of respiratory complexes. This demonstrates the power of this technique to better understand the morpho-functional correlates of mitochondrial dynamics which might be critical to understanding disease. Here, we demonstrated that MitoTouch can capture subtle differences in mitochondrial organization between skin tumor cells and their normal counterparts, suggesting that our image analysis software may be useful for identifying and quantifying tumor cell phenotypes.

## Conclusions

Because a growing body of literature points to mitochondria as a key organelle associated with diseases [3, 13–21, 69] and targeted by environmental pollutants [62, 83–87], quantitative imaging tools like MitoTouch has the potential to open new fields both in pathology and in mechanistic toxicology for various target tissues and species (in an ‘One Health’ perspective). Although our procedure was mainly tested on live human skin cells, live cell images (taken through fluorescence imaging techniques like confocal microscopy or two-photon excitation microscopy) can be collected from virtually any type of adherent cells before segmentation and automatic processing. Last, thanks to the availability of specific fluorescent probes, it might be interesting to adapt our procedure and tools to study the morphology and dynamics of other organelles including lysosomes, the Golgi apparatus and the endoplasmic reticulum [88, 89].

## Supporting information

S1 File Raw results and plots. The raw data are accessible at https://doi.org/10.48579/PRO/ROSLGY. XLS_raw_data: content of the datasets used for statistical analysis; BERG_plots: plots made with R for the experiments with the natural ingredient Bergaphen-15; CANCER_plots: plots made with R for the experiments using human normal versus cancer fibroblasts; NHDF_NHEK_plots: plots made with R for the experiments using normal human dermal fibroblasts and normal human epidermal keratinocytes; TOXICANTS_plots: plots made with R for the experiments using toxicant-treated cells; UVB_plots: plots made with R for the experiments using UVB-irradiated cells.
(PDF)

S2 File MitoGrid. The 31 morphometric parameters defined for analyzing the mitochondrial network in the cell allow us to establish the different configuration possibilities of this network (M1: reference; M2: fusion; M3: branching; M4: compaction; M5: dislocation; M6: total or partial fission; M7: total or partial swollen), its position in the cell (I1: reference; I2: pericortical; I3: perinuclear), and the resulting cellular form (C1: reference; C2: retraction; C3: spreading; C4: compaction; C5: star-like; C6: CellMask intensity). Theoretical MitoSpider plots were created by varying the parameters of shape, connectivity, size, density, texture, and abundance, and were associated with different predetermined configurations. The violet background of the Spiderplot indicates the active state of the illustration, and the corresponding parameters are shown in orange upon decrease and in brown upon increase.
(PDF)

S3 File MitoSpider-Board and MitoSigil-Board. All possible theoretical MitoSpider plots are represented. All schematic representations in the form of a MitoSigil, each corresponding to a theoretical Spiderplot, have been aligned on these sheets.
(PDF)

S4 File Comparison of MitoTouch with manual and semi-automatic quantification techniques. Human osteosarcoma U2OS cells were treated with vehicle (0,5% DMSO) or 20μM CCCP for 2h 30 min before staining with with 2.5μg/mL Hoechst 33342, 5 μg/mL Cell Mask Green and 250nM Mitotracker Deep Red and confocal imaging. Manual analysis: A-D; Semi-automatic analysis: E-H; MitoTouch analysis: I-K. **(A)** The framed area (956x955 pixels) equivalent to 19.6% of the original image was analyzed (corresponding to a total of 6 cells). **(B)** MitoTouch’s segmentation in the same area for visual comparison. **(C)** Manual delineation of mitochondria (yellow segments) in 6 different cells. A total of 362 lines (FreeHand lines in ImageJ) were defined manually in 720s. Automatic quantification in this zone detected 417 mitochondria in 16s (time saving factor x45). Note that mitochondrial clusters cannot be delineated. **(D)** Quantification of five parameters (in contrast to the 31 parameters computed by MitoTouch): Area, Length, Intensity Mean, Min, Max using ImageJ’s mesure function. **(E)** Macro description steps and analyzed features (a). Specification of recorded measurements: Area, Shape descriptors (Circularity, Aspect Ratio, Roundness, Solidity) & Integrated Density (b). Settings adjustments for minimum size and maximum pixel area size and roundness value (c). **(F)** Original confocal image of a single cell (a). Threshold adjustment with dark background and red particles (b). Mask conversion: output is a binary image black and white, with foreground 255 and background 0, using an inverted LUT (c). Measurements and particle analysis (d). **(G)** U2OS cells treated with vehicle (0,5% DMSO) (a-c) or 20μM CCCP (d-f). Pre-processing step (mitochondria mask: b, e). Quantification step (c, f). **(H)** MitoSpider plot representation. Seven parameters were computed (in contrast to the 31 parameters offered by MitoTouch): Area, Circularity, Integrated Density (IntDen: Area multiplied by the Mean gray value), Raw Integrated Density (RawIntDen: the sum of all pixel values in the region of interest), Aspect Ratio, Roundness, Solidity. Automatic analysis of 2 images (containing ~ 25 cells by field) took around 10s (number of mitochondria analyzed in the control condition: 1860; number of mitochondria analyzed in the treated condition: 2896). Analysis of the framed area defined in (A) took around 2s. Mitochondrial roundness and circularity were increased in CCCP treated-cells whereas mitochondrial area was decreased, indicative of mitochondrial fragmentation. Note that branching parameters for network analysis were not available using this method, in contrast to MitoTouch. **(I)** Confocal images of U2OS cells treated with vehicle (0,5% DMSO) (a-f) or 20μM CCCP (g-l) and stained with Hoechst 33342 (a, g), Cell Mask Green (b, h) and Mitotracker Deep Red (c, i). Masks corresponding to Nuclei segmentation (Nuclei Threshold 2) (d-j), Cell segmentation (Cell Threshold 1) (e-k) and Mitochondrial clusters (Mito Threshold 1) (f-l). **(J)** Statistical analysis (t-test) and graphical representation in the form of a MitoSpider Plot (see main text for details). The 31 parameters measured by the MitoTouch software are reported after normalization on a MitoSpider graph. Features are grouped into 5 categories including *Abundance*, *Shape*, *Connectivity*, *Size*, *Density* and *Texture*. Significant differences from basal (vehicle: green circle) and CCCP (treated: blue circle) are notified by small (*:p<0.05), medium (**:p<0.01) or large (***:p<0.001) circles (t-test). **(K)** Schematic representation (MitoSigil) reflecting the organization of the mitochondria, their positioning in the cell as well as the global cellular shape (see S2 and S3 Files for details). A total of 6 images (3 in each tested condition, containing approximately 25 cells by field) were automatically analyzed in 169 s (including the image segmentation and parameter computation steps). Results indicate that CCCP exposure triggers mitochondrial network dislocation and mitochondrial fragmentation (increases in mitochondrial fission: +86%, mitochondrial compaction: +80%, mitochondrial dislocation: +55%) as well as repositioning of the mitochondrial network closer to the nucleus (+66%) in cells that appear smaller and rounder (cell compaction: +50%; cell retraction: +50%). In addition to being faster, automated analysis using MitoTouch is thus more accurate and exhaustive than manual or semi-automatic analysis.
(PDF)
