# Supplementary material for: Quantitative imaging and semiotic phenotyping of mitochondrial network morphology in live human cells
Source: PLoS One. 2024 Mar 28;19(3):e0301372. doi: 10.1371/journal.pone.0301372 (PMC10977735; doi:10.1371/journal.pone.0301372)
Supplement: S1 File — The raw data are accessible at https://doi.org/10.48579/PRO/ROSLGY. XLS_raw_data: content of the datasets used for statistical analysis; BERG_plots: plots made with R for the experiments with the natural ingredient Bergaphen-15; CANCER_plots: plots made with R for the experiments using human normal versus cancer fibroblasts; NHDF_NHEK_plots: plots made with R for the experiments using normal human dermal fibroblasts and normal human epidermal keratinocytes; TOXICANTS_plots: plots made with R for the experiments using toxicant-treated cells; UVB_plots: plots made with R for the experiments using UVB-irradiated cells. (PDF) [file pone.0301372.s001.pdf]

S1 File. Raw results and plots.

The raw data are accessible at https://doi.org/10.48579/PRO/ROSLGY. XLS_raw_data: content of the datasets used for statistical analysis; BERG_plots: plots made with R for the experiments with the natural ingredient Bergaphen-15; CANCER_plots: plots made with R for the experiments using human normal versus cancer fibroblasts; NHDF_NHEK_plots: plots made with R for the experiments using normal human dermal fibroblasts and normal human epidermal keratinocytes; TOXICANTS_plots: plots made with R for the experiments using toxicant-treated cells; UVB_plots: plots made with R for the experiments using UVB-irradiated cells.
