# Supplementary material for: Quantitative imaging and semiotic phenotyping of mitochondrial network morphology in live human cells
Source: PLoS One. 2024 Mar 28;19(3):e0301372. doi: 10.1371/journal.pone.0301372 (PMC10977735; doi:10.1371/journal.pone.0301372)
Supplement: S2 File — The 31 morphometric parameters defined for analyzing the mitochondrial network in the cell allow us to establish the different configuration possibilities of this network (M1: reference; M2: fusion; M3: branching; M4: compaction; M5: dislocation; M6: total or partial fission; M7: total or partial swollen), its position in the cell (I1: reference; I2: pericortical; I3: perinuclear), and the resulting cellular form (C1: reference; C2: retraction; C3: spreading; C4: compaction; C5: star-like; C6: CellMask intensity). Theoretical MitoSpider plots were created by varying the parameters of shape, connectivity, size, density, texture, and abundance, and were associated with different predetermined configurations. The violet background of the Spiderplot indicates the active state of the illustration, and the corresponding parameters are shown in orange upon decrease and in brown upon increase. (PDF) [file pone.0301372.s002.pdf]

S2 File. MitoGrid.

The 31 morphometric parameters defined for analyzing the mitochondrial network in the cell allow us to establish the different configuration possibilities of this network (M1: reference; M2: fusion; M3: branching; M4: compaction; M5: dislocation; M6: total or partial fission; M7: total or partial swollen), its position in the cell (I1: reference; I2: pericortical; I3: perinuclear), and the resulting cellular form (C1: reference; C2: retraction; C3: spreading; C4: compaction; C5: star-like; C6: CellMask intensity). Theoretical MitoSpider plots were created by varying the parameters of shape, connectivity, size, density, texture, and abundance, and were associated with different predetermined configurations. The violet background of the Spiderplot indicates the active state of the illustration, and the corresponding parameters are shown in orange upon decrease and in brown upon increase.

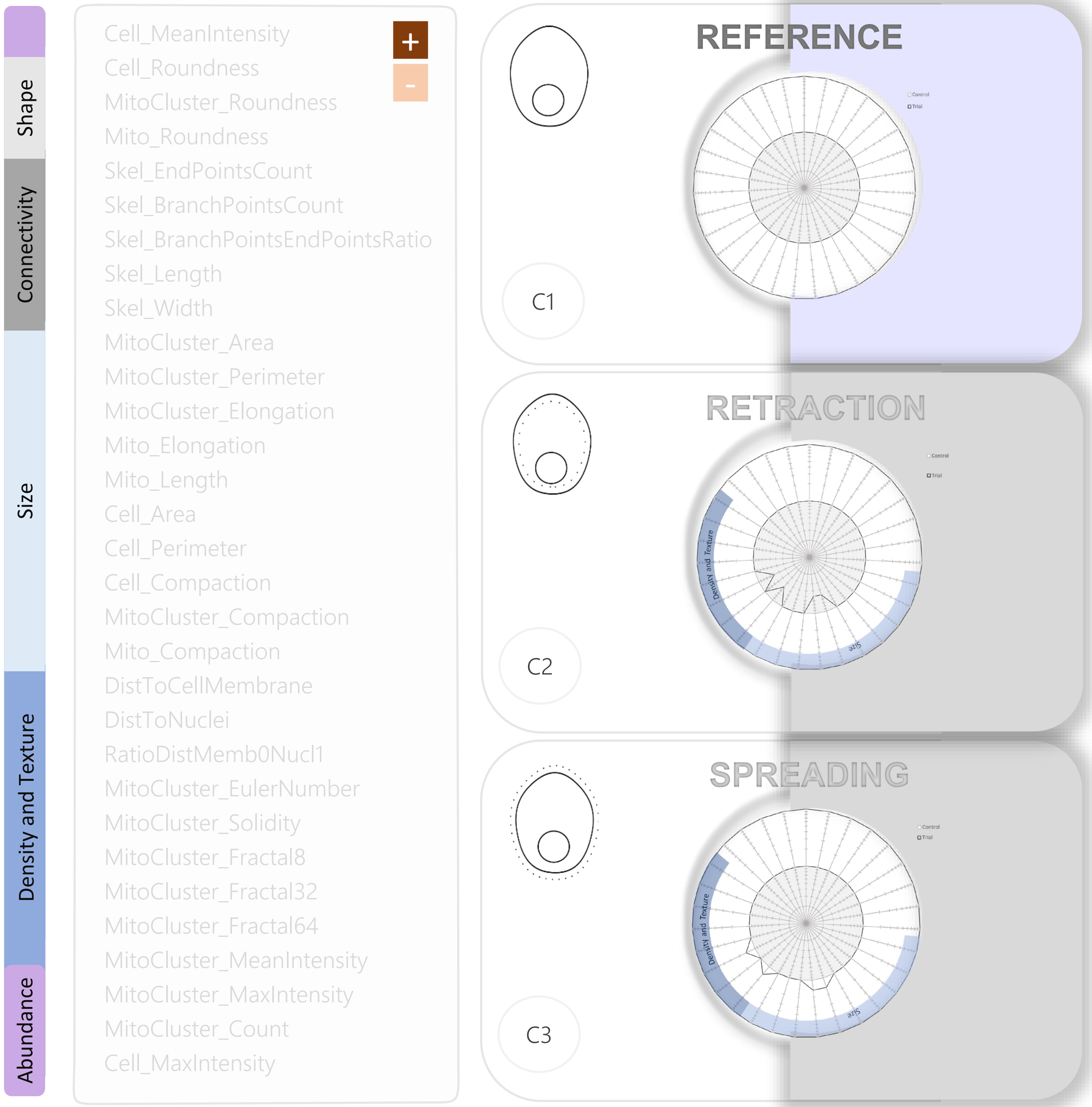
Shape
Connectivity
Size
Density and Texture
Abundance
Cell_MeanIntensity
Cell_Roundness
MitoCluster_Roundness
Mito_Roundness
Skel_EndPointsCount
Skel_BranchPointsCount
Skel_BranchPointsEndPointsRatio
Skel_Length
Skel_Width
MitoCluster_Area
MitoCluster_Perimeter
MitoCluster_Elongation
Mito_Elongation
Mito_Length
Cell_Area
Cell_Perimeter
Cell_Compaction
MitoCluster_Compaction
Mito_Compaction
DistToCellMembrane
DistToNuclei
RatioDistMemb0Nucl1
MitoCluster_EulerNumber
MitoCluster_Solidity
MitoCluster_Fractal8
MitoCluster_Fractal32
MitoCluster_Fractal64
MitoCluster_MeanIntensity
MitoCluster_MaxIntensity
MitoCluster_Count
Cell_MaxIntensity
+
-
REFERENCE
C1
RETRACTION
C2
Density and Texture
Size
SPREADING
C3
Density and Texture
Size

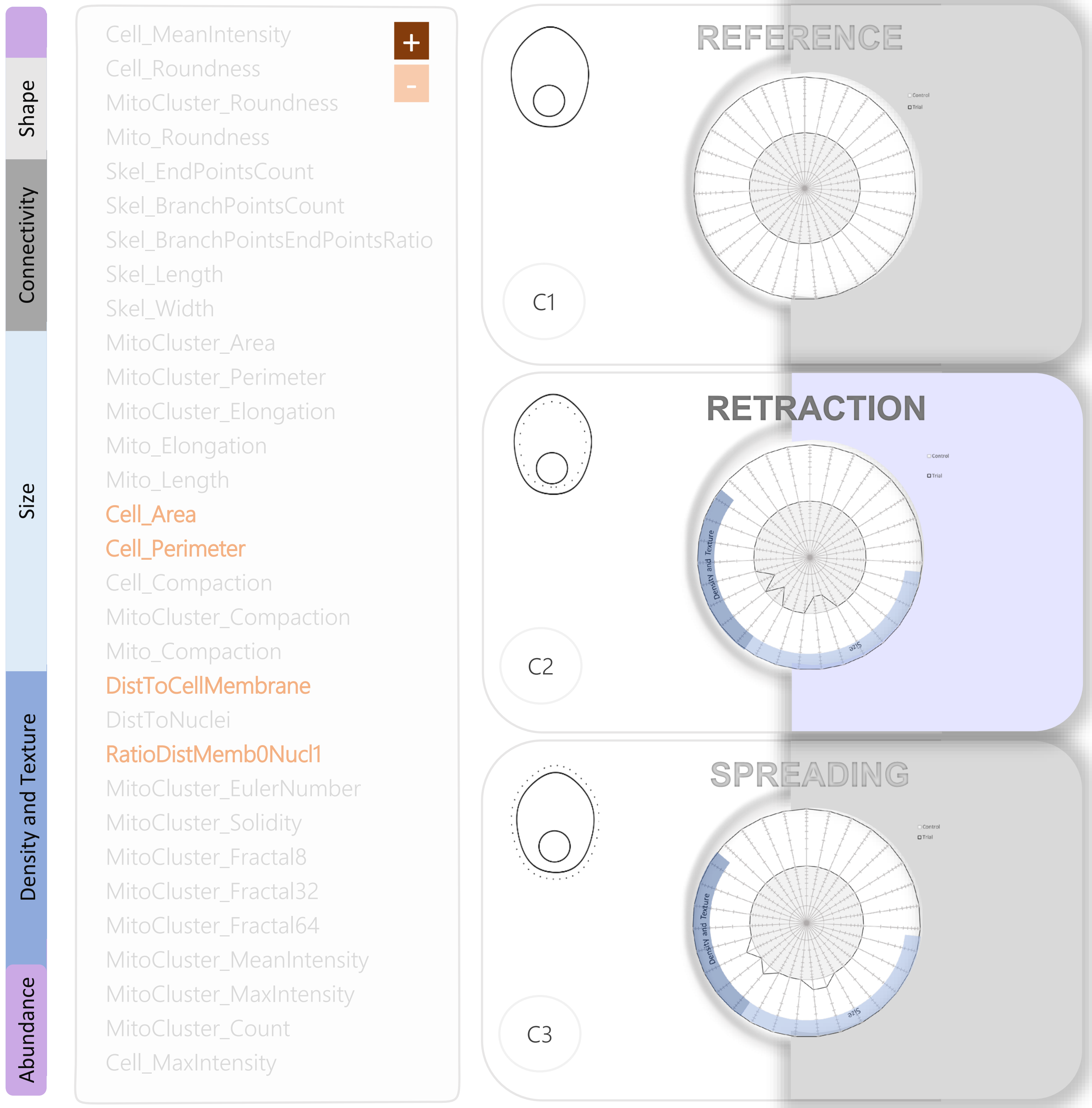
Shape
Connectivity
Size
Density and Texture
Abundance
Cell_MeanIntensity
Cell_Roundness
MitoCluster_Roundness
Mito_Roundness
Skel_EndPointsCount
Skel_BranchPointsCount
Skel_BranchPointsEndPointsRatio
Skel_Length
Skel_Width
MitoCluster_Area
MitoCluster_Perimeter
MitoCluster_Elongation
Mito_Elongation
Mito_Length
Cell_Area
Cell_Perimeter
Cell_Compaction
MitoCluster_Compaction
Mito_Compaction
DistToCellMembrane
DistToNuclei
RatioDistMemb0Nucl1
MitoCluster_EulerNumber
MitoCluster_Solidity
MitoCluster_Fractal8
MitoCluster_Fractal32
MitoCluster_Fractal64
MitoCluster_MeanIntensity
MitoCluster_MaxIntensity
MitoCluster_Count
Cell_MaxIntensity
+
-
REFERENCE
C1
RETRACTION
Density and Texture
Size
C2
SPREADING
Density and Texture
Size
C3

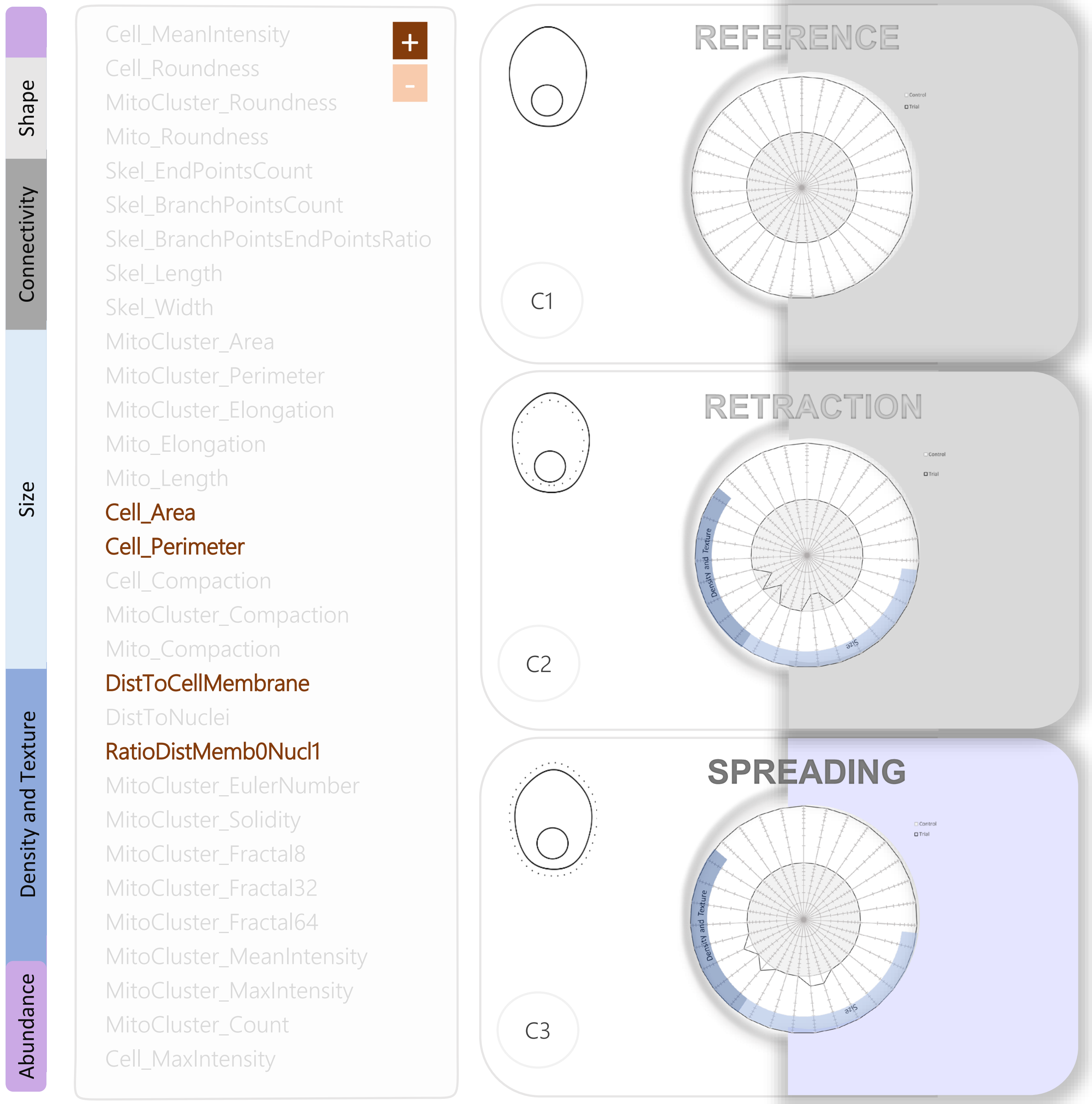
Shape
Connectivity
Size
Density and Texture
Abundance
Cell_MeanIntensity
Cell_Roundness
MitoCluster_Roundness
Mito_Roundness
Skel_EndPointsCount
Skel_BranchPointsCount
Skel_BranchPointsEndPointsRatio
Skel_Length
Skel_Width
MitoCluster_Area
MitoCluster_Perimeter
MitoCluster_Elongation
Mito_Elongation
Mito_Length
Cell_Area
Cell_Perimeter
Cell_Compaction
MitoCluster_Compaction
Mito_Compaction
DistToCellMembrane
DistToNuclei
RatioDistMemb0Nucl1
MitoCluster_EulerNumber
MitoCluster_Solidity
MitoCluster_Fractal8
MitoCluster_Fractal32
MitoCluster_Fractal64
MitoCluster_MeanIntensity
MitoCluster_MaxIntensity
MitoCluster_Count
Cell_MaxIntensity
+
-
REFERENCE
C1
RETRACTION
C2
SPREADING
C3

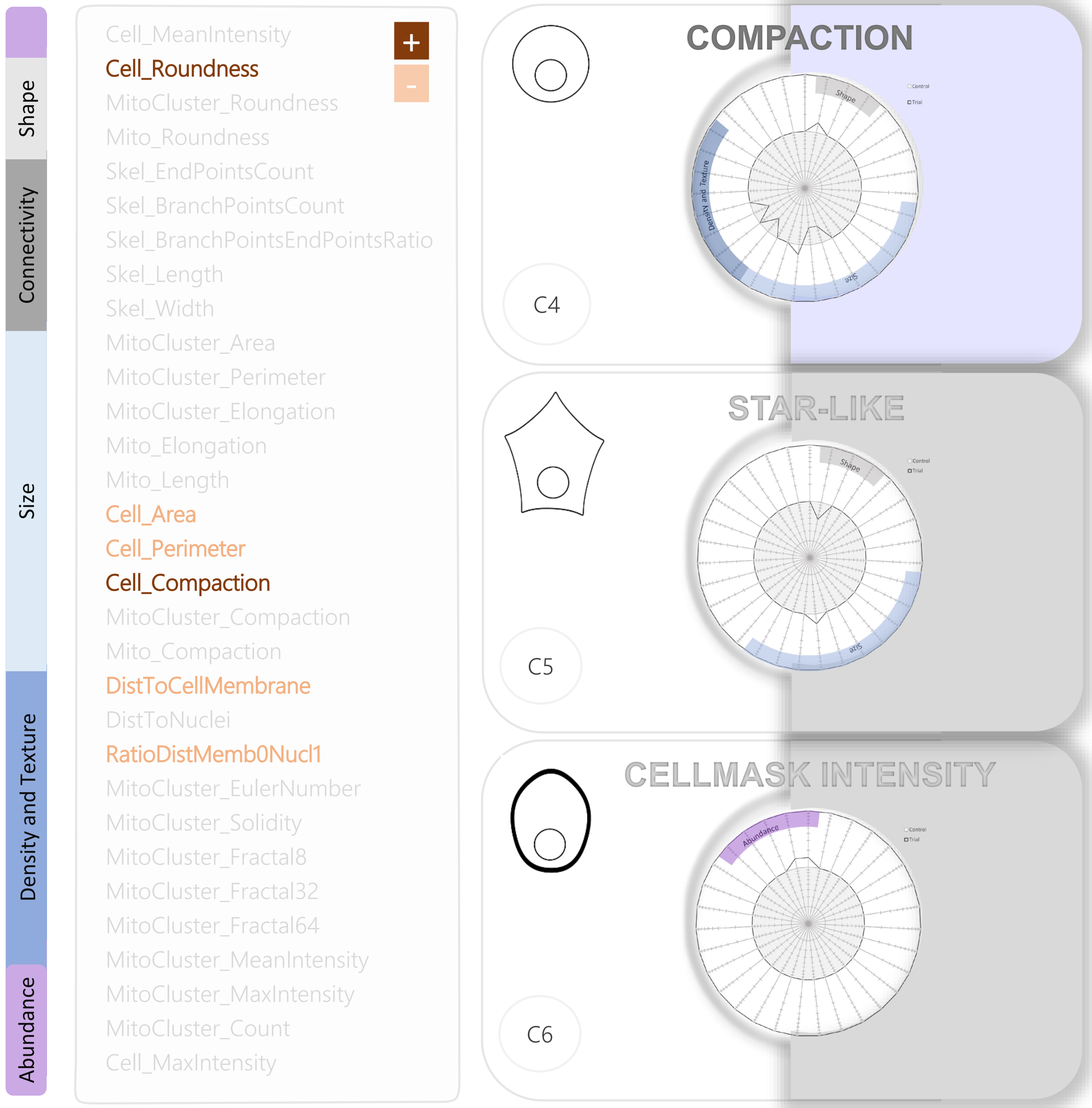
Shape
Connectivity
Size
Density and Texture
Abundance
Cell_MeanIntensity
Cell_Roundness
MitoCluster_Roundness
Mito_Roundness
Skel_EndPointsCount
Skel_BranchPointsCount
Skel_BranchPointsEndPointsRatio
Skel_Length
Skel_Width
MitoCluster_Area
MitoCluster_Perimeter
MitoCluster_Elongation
Mito_Elongation
Mito_Length
Cell_Area
Cell_Perimeter
Cell_Compaction
MitoCluster_Compaction
Mito_Compaction
DistToCellMembrane
DistToNuclei
RatioDistMemb0Nucl1
MitoCluster_EulerNumber
MitoCluster_Solidity
MitoCluster_Fractal8
MitoCluster_Fractal32
MitoCluster_Fractal64
MitoCluster_MeanIntensity
MitoCluster_MaxIntensity
MitoCluster_Count
Cell_MaxIntensity
+
-
COMPACTION
C4
STAR-LIKE
C5
CELLMASK INTENSITY
C6

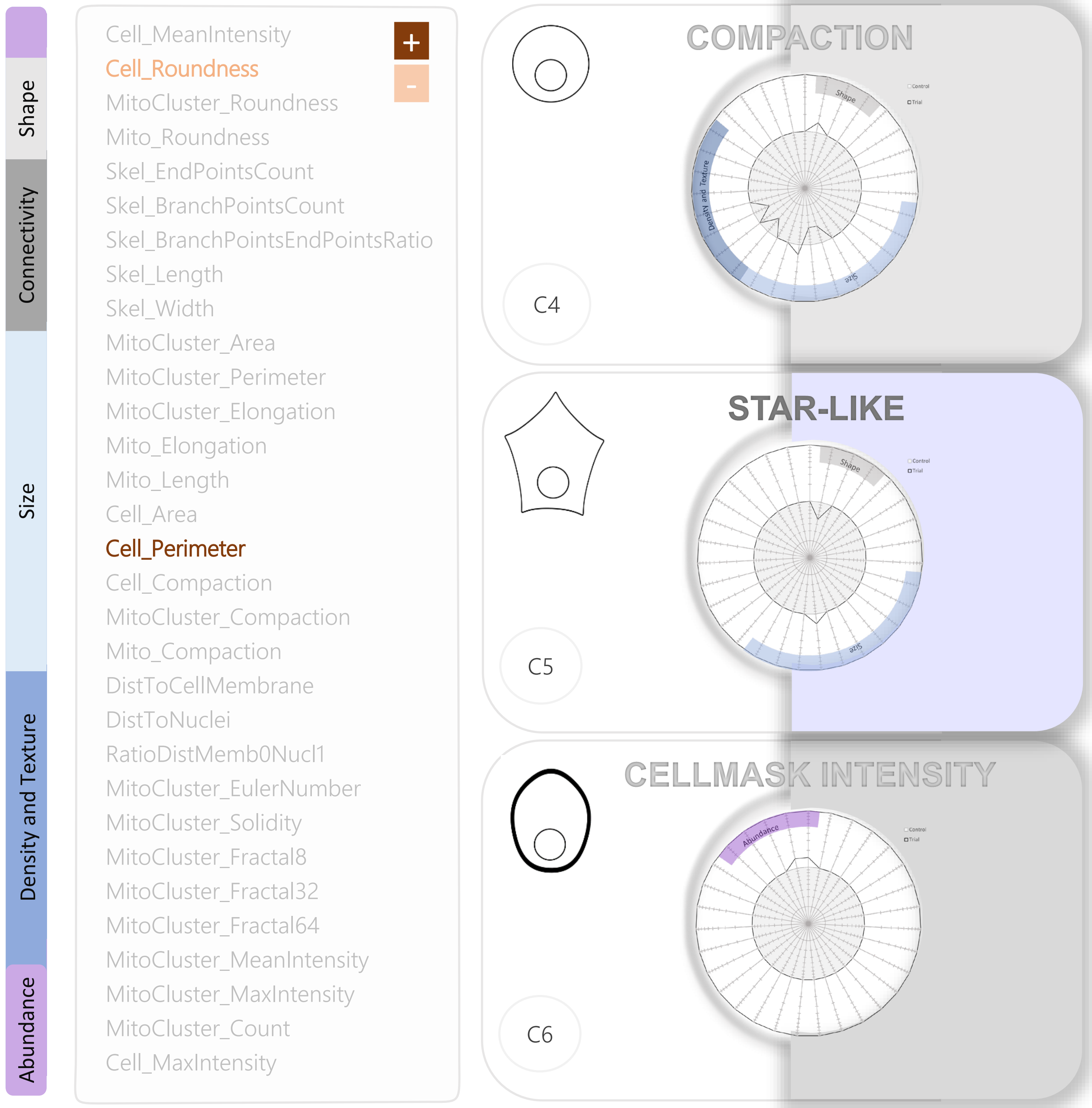
Shape
Connectivity
Size
Density and Texture
Abundance
Cell_MeanIntensity
Cell_Roundness
MitoCluster_Roundness
Mito_Roundness
Skel_EndPointsCount
Skel_BranchPointsCount
Skel_BranchPointsEndPointsRatio
Skel_Length
Skel_Width
MitoCluster_Area
MitoCluster_Perimeter
MitoCluster_Elongation
Mito_Elongation
Mito_Length
Cell_Area
Cell_Perimeter
Cell_Compaction
MitoCluster_Compaction
Mito_Compaction
DistToCellMembrane
DistToNuclei
RatioDistMemb0Nucl1
MitoCluster_EulerNumber
MitoCluster_Solidity
MitoCluster_Fractal8
MitoCluster_Fractal32
MitoCluster_Fractal64
MitoCluster_MeanIntensity
MitoCluster_MaxIntensity
MitoCluster_Count
Cell_MaxIntensity
+
-
COMPACTION
C4
STAR-LIKE
C5
CELLMASK INTENSITY
C6

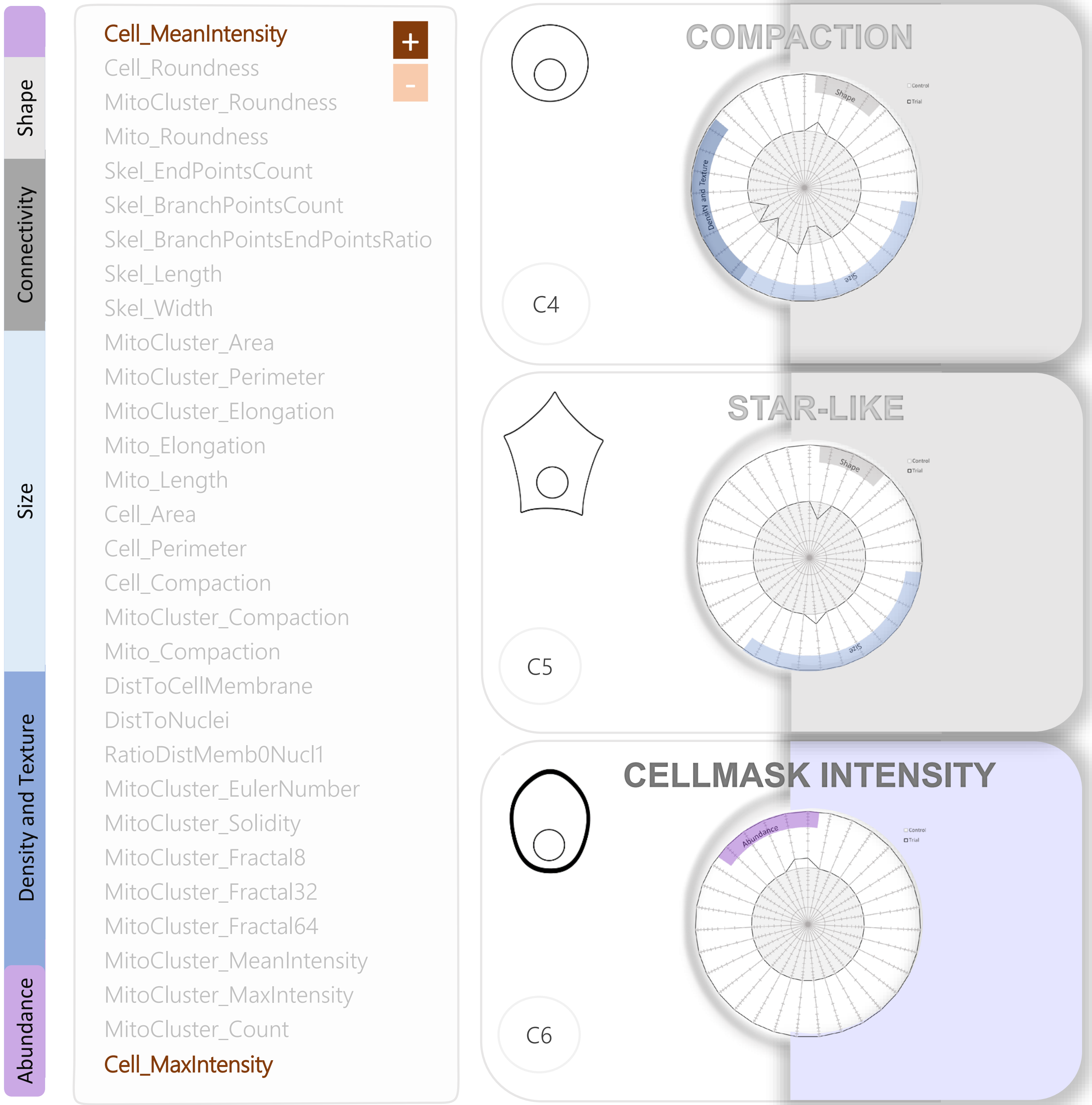
Shape
Connectivity
Size
Density and Texture
Abundance
Cell_MeanIntensity
+
-
Cell_Roundness
MitoCluster_Roundness
Mito_Roundness
Skel_EndPointsCount
Skel_BranchPointsCount
Skel_BranchPointsEndPointsRatio
Skel_Length
Skel_Width
MitoCluster_Area
MitoCluster_Perimeter
MitoCluster_Elongation
Mito_Elongation
Mito_Length
Cell_Area
Cell_Perimeter
Cell_Compaction
MitoCluster_Compaction
Mito_Compaction
DistToCellMembrane
DistToNuclei
RatioDistMemb0Nucl1
MitoCluster_EulerNumber
MitoCluster_Solidity
MitoCluster_Fractal8
MitoCluster_Fractal32
MitoCluster_Fractal64
MitoCluster_MeanIntensity
MitoCluster_MaxIntensity
MitoCluster_Count
Cell_MaxIntensity
COMPACTION
C4
STAR-LIKE
C5
CELLMASK INTENSITY
C6

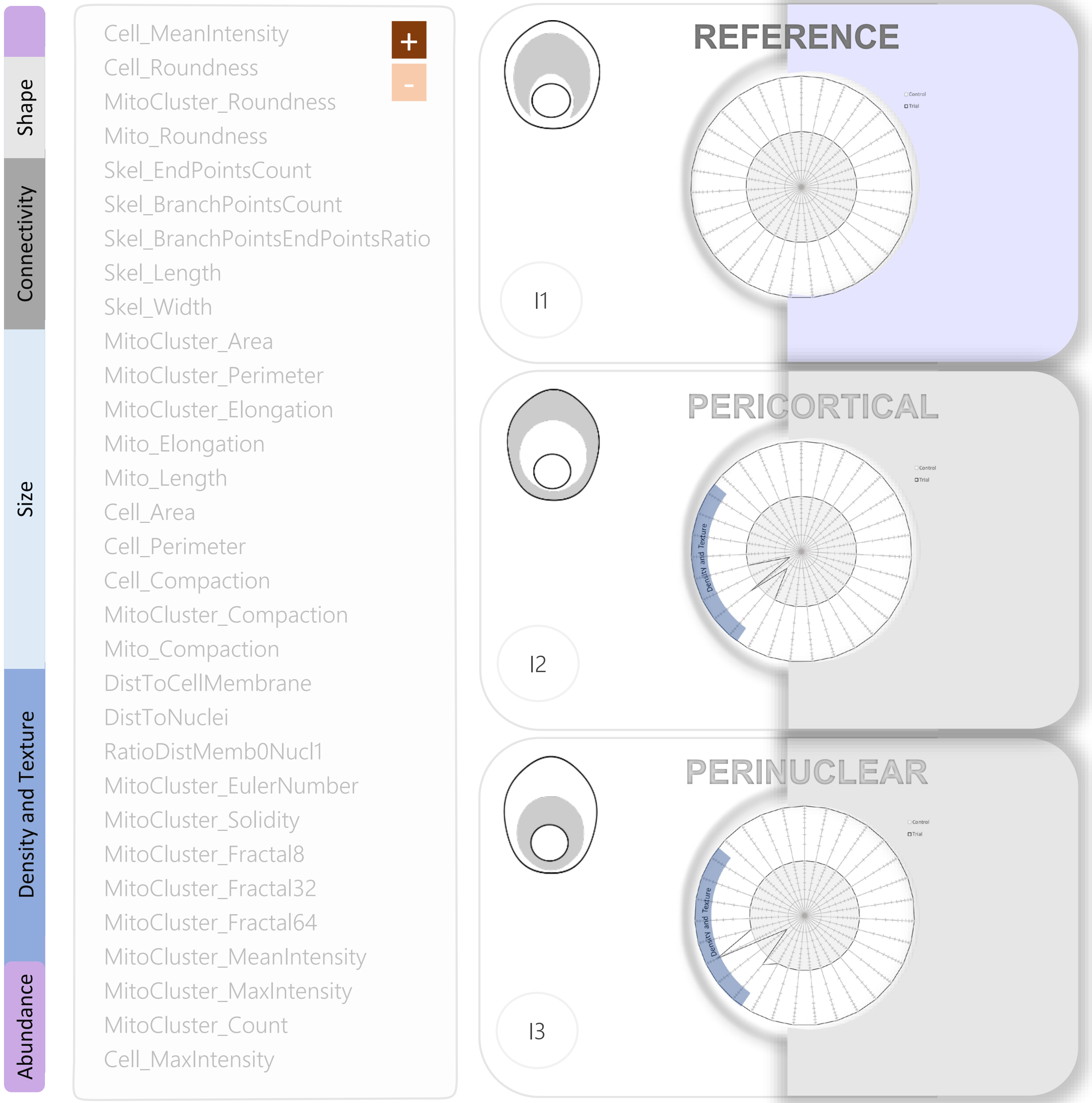
Shape
Connectivity
Size
Density and Texture
Abundance
Cell_MeanIntensity
Cell_Roundness
MitoCluster_Roundness
Mito_Roundness
Skel_EndPointsCount
Skel_BranchPointsCount
Skel_BranchPointsEndPointsRatio
Skel_Length
Skel_Width
MitoCluster_Area
MitoCluster_Perimeter
MitoCluster_Elongation
Mito_Elongation
Mito_Length
Cell_Area
Cell_Perimeter
Cell_Compaction
MitoCluster_Compaction
Mito_Compaction
DistToCellMembrane
DistToNuclei
RatioDistMemb0Nucl1
MitoCluster_EulerNumber
MitoCluster_Solidity
MitoCluster_Fractal8
MitoCluster_Fractal32
MitoCluster_Fractal64
MitoCluster_MeanIntensity
MitoCluster_MaxIntensity
MitoCluster_Count
Cell_MaxIntensity
+
-
REFERENCE
I1
PERICORTICAL
I2
PERINUCLEAR
I3

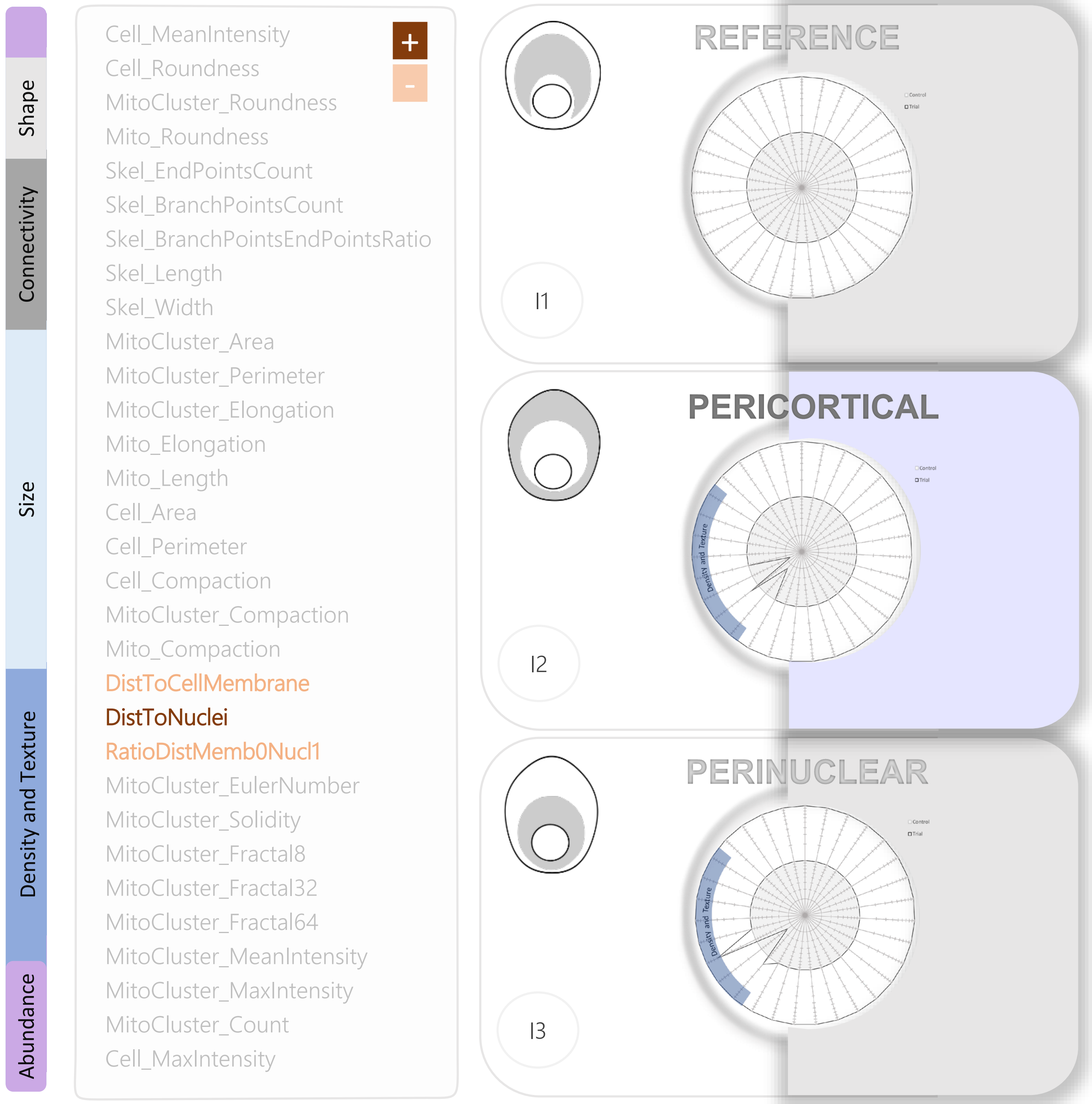
Shape
Connectivity
Size
Density and Texture
Abundance
Cell_MeanIntensity
Cell_Roundness
MitoCluster_Roundness
Mito_Roundness
Skel_EndPointsCount
Skel_BranchPointsCount
Skel_BranchPointsEndPointsRatio
Skel_Length
Skel_Width
MitoCluster_Area
MitoCluster_Perimeter
MitoCluster_Elongation
Mito_Elongation
Mito_Length
Cell_Area
Cell_Perimeter
Cell_Compaction
MitoCluster_Compaction
Mito_Compaction
DistToCellMembrane
DistToNuclei
RatioDistMemb0Nucl1
MitoCluster_EulerNumber
MitoCluster_Solidity
MitoCluster_Fractal8
MitoCluster_Fractal32
MitoCluster_Fractal64
MitoCluster_MeanIntensity
MitoCluster_MaxIntensity
MitoCluster_Count
Cell_MaxIntensity
+
-
REFERENCE
I1
PERICORTICAL
I2
PERINUCLEAR
I3

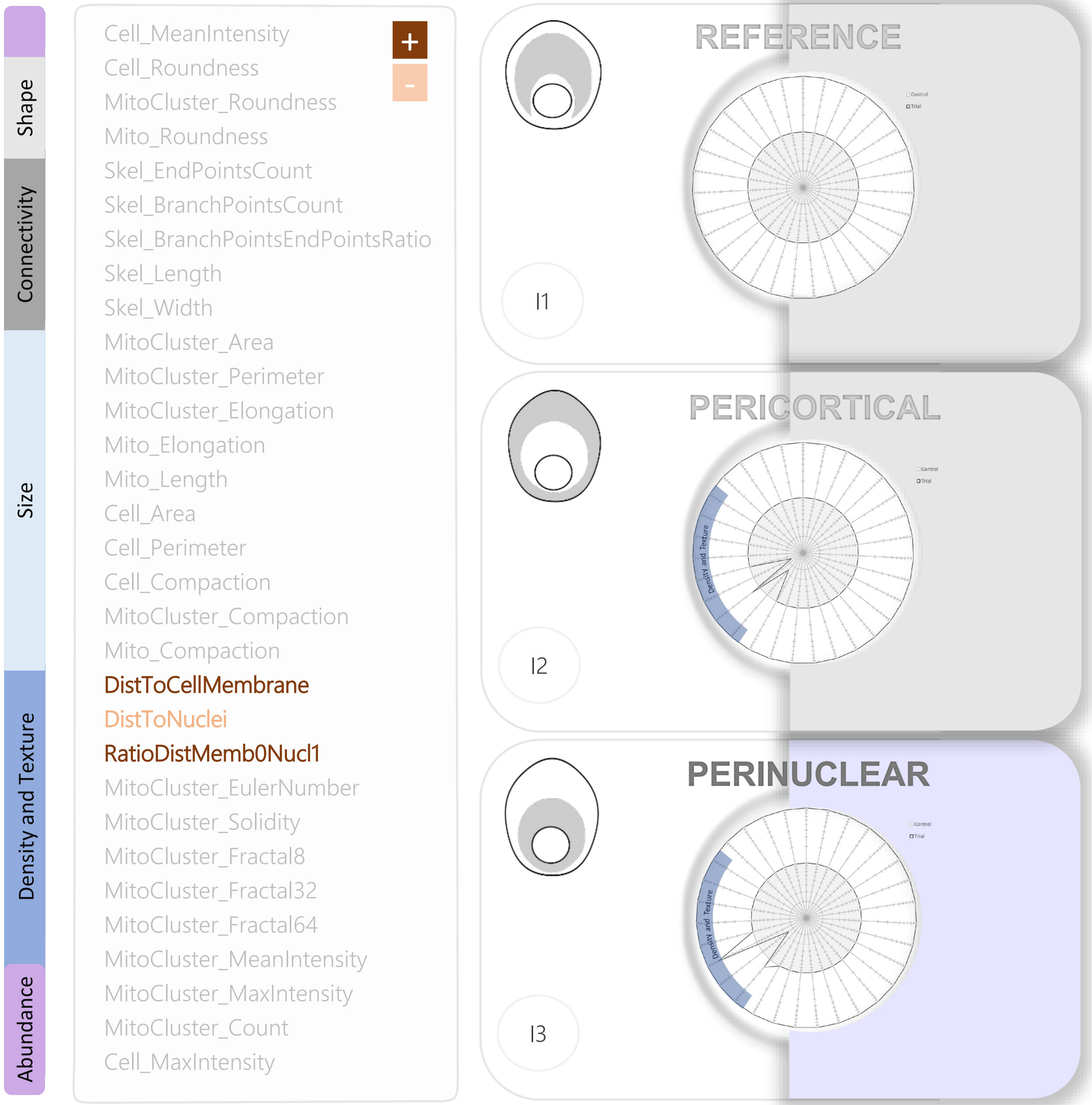
Shape
Connectivity
Size
Density and Texture
Abundance
Cell_MeanIntensity
Cell_Roundness
MitoCluster_Roundness
Mito_Roundness
Skel_EndPointsCount
Skel_BranchPointsCount
Skel_BranchPointsEndPointsRatio
Skel_Length
Skel_Width
MitoCluster_Area
MitoCluster_Perimeter
MitoCluster_Elongation
Mito_Elongation
Mito_Length
Cell_Area
Cell_Perimeter
Cell_Compaction
MitoCluster_Compaction
Mito_Compaction
DistToCellMembrane
DistToNuclei
RatioDistMemb0Nucl1
MitoCluster_EulerNumber
MitoCluster_Solidity
MitoCluster_Fractal8
MitoCluster_Fractal32
MitoCluster_Fractal64
MitoCluster_MeanIntensity
MitoCluster_MaxIntensity
MitoCluster_Count
Cell_MaxIntensity
+
-
REFERENCE
I1
PERICORTICAL
I2
PERINUCLEAR
I3

Shape
Connectivity
Size
Density and Texture
Abundance

+
-

Cell_MeanIntensity
Cell_Roundness
MitoCluster_Roundness
Mito_Roundness
Skel_EndPointsCount
Skel_BranchPointsCount
Skel_BranchPointsEndPointsRatio
Skel_Length
Skel_Width
MitoCluster_Area
MitoCluster_Perimeter
MitoCluster_Elongation
Mito_Elongation
Mito_Length
Cell_Area
Cell_Perimeter
Cell_Compaction
MitoCluster_Compaction
Mito_Compaction
DistToCellMembrane
DistToNuclei
RatioDistMemb0Nucl1
MitoCluster_EulerNumber
MitoCluster_Solidity
MitoCluster_Fractal8
MitoCluster_Fractal32
MitoCluster_Fractal64
MitoCluster_MeanIntensity
MitoCluster_MaxIntensity
MitoCluster_Count
Cell_MaxIntensity

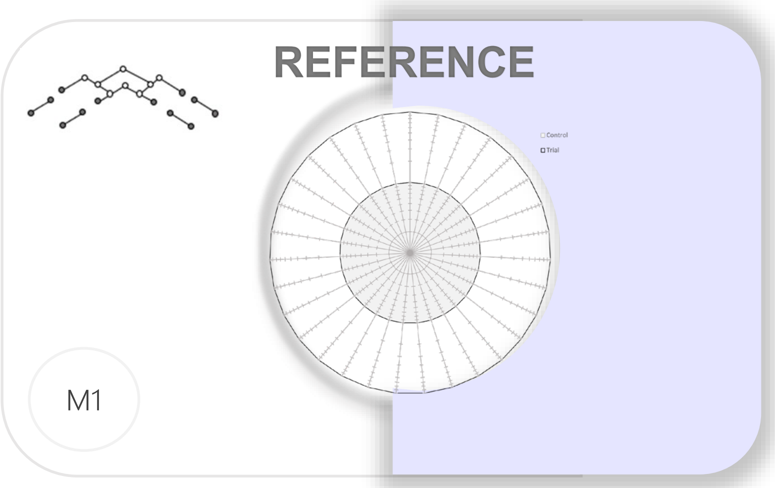

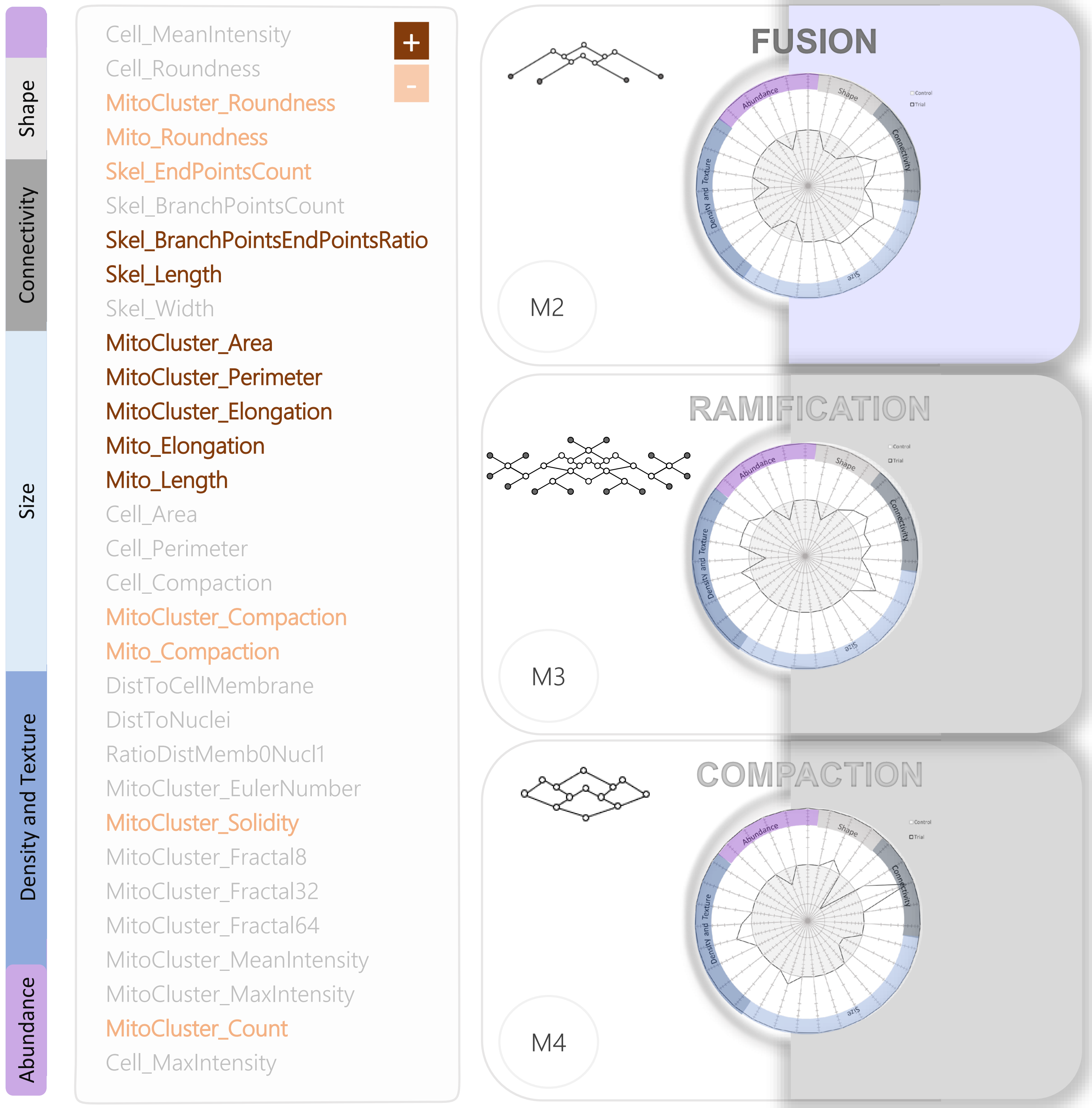
Shape
Connectivity
Size
Density and Texture
Abundance
Cell_MeanIntensity
Cell_Roundness
MitoCluster_Roundness
Mito_Roundness
Skel_EndPointsCount
Skel_BranchPointsCount
Skel_BranchPointsEndPointsRatio
Skel_Length
Skel_Width
MitoCluster_Area
MitoCluster_Perimeter
MitoCluster_Elongation
Mito_Elongation
Mito_Length
Cell_Area
Cell_Perimeter
Cell_Compaction
MitoCluster_Compaction
Mito_Compaction
DistToCellMembrane
DistToNuclei
RatioDistMemb0Nucl1
MitoCluster_EulerNumber
MitoCluster_Solidity
MitoCluster_Fractal8
MitoCluster_Fractal32
MitoCluster_Fractal64
MitoCluster_MeanIntensity
MitoCluster_MaxIntensity
MitoCluster_Count
Cell_MaxIntensity
+
-
FUSION
M2
RAMIFICATION
M3
COMPACTION
M4

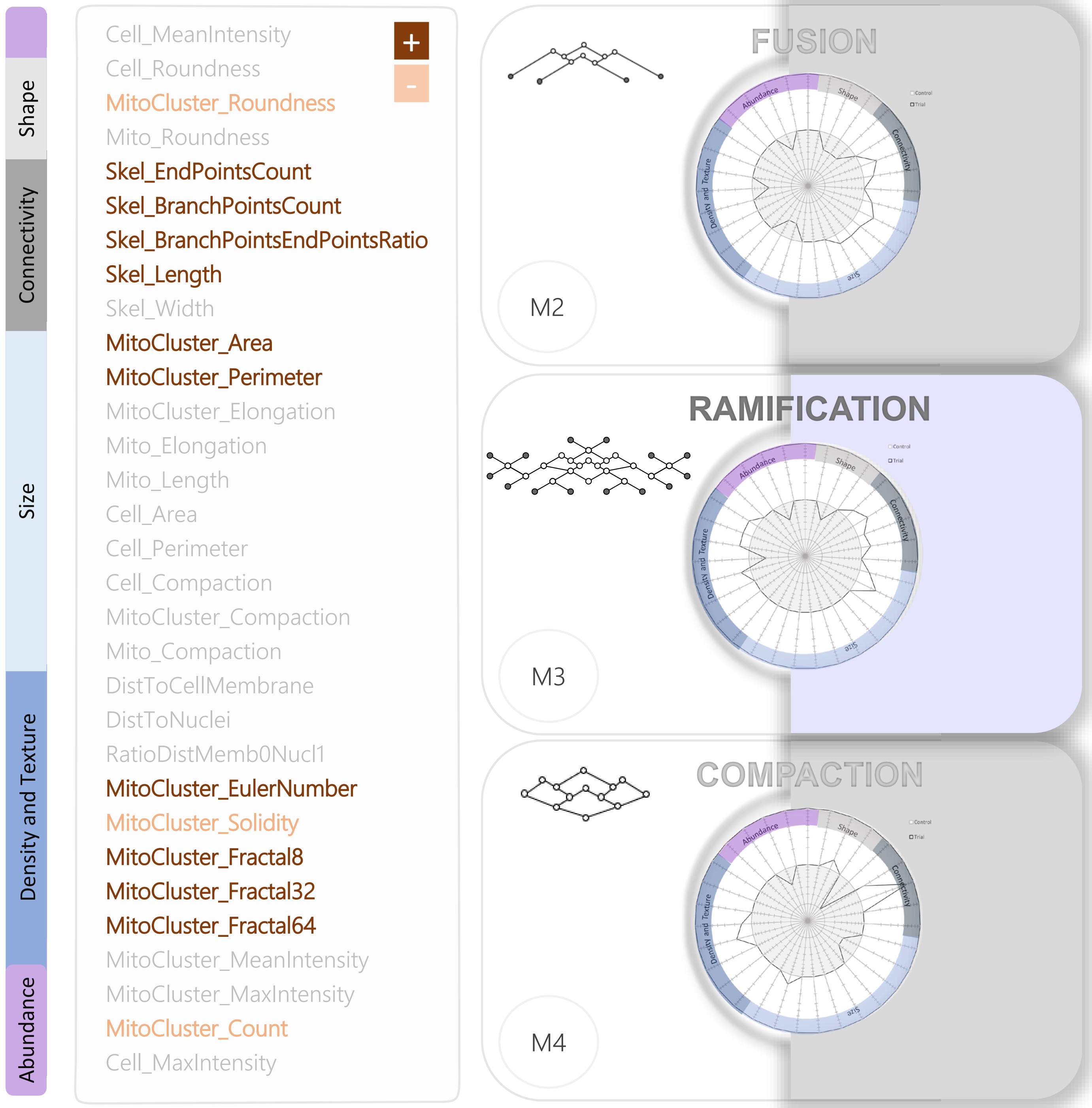

Shape
Connectivity
Size
Density and Texture
Abundance
Cell_MeanIntensity
Cell_Roundness
MitoCluster_Roundness
Mito_Roundness
Skel_EndPointsCount
Skel_BranchPointsCount
Skel_BranchPointsEndPointsRatio
Skel_Length
Skel_Width
MitoCluster_Area
MitoCluster_Perimeter
MitoCluster_Elongation
Mito_Elongation
Mito_Length
Cell_Area
Cell_Perimeter
Cell_Compaction
MitoCluster_Compaction
Mito_Compaction
DistToCellMembrane
DistToNuclei
RatioDistMemb0Nucl1
MitoCluster_EulerNumber
MitoCluster_Solidity
MitoCluster_Fractal8
MitoCluster_Fractal32
MitoCluster_Fractal64
MitoCluster_MeanIntensity
MitoCluster_MaxIntensity
MitoCluster_Count
Cell_MaxIntensity
+
-
FUSION
M2
RAMIFICATION
M3
COMPACTION
M4

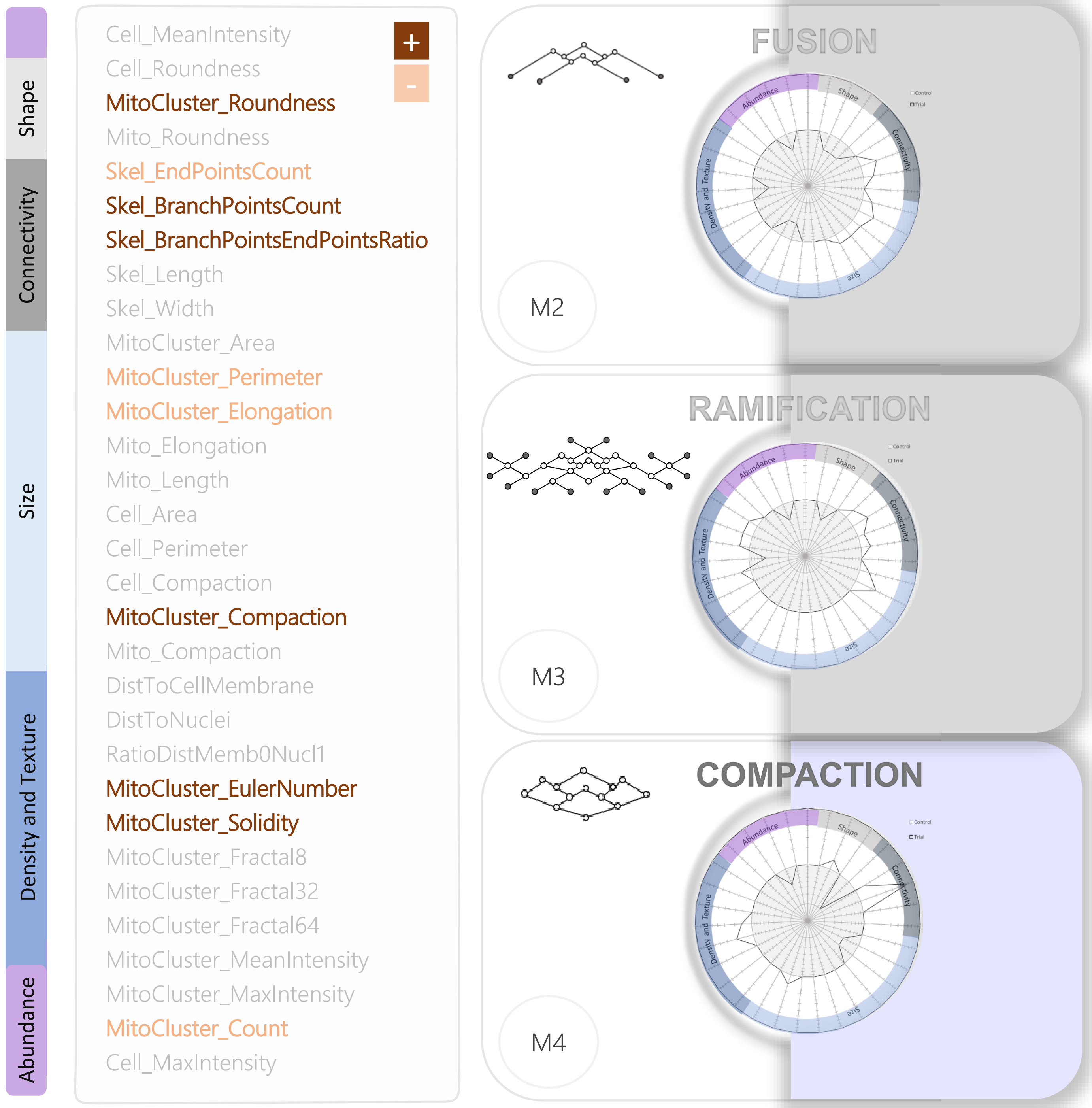
Shape
Connectivity
Size
Density and Texture
Abundance
Cell_MeanIntensity
Cell_Roundness
MitoCluster_Roundness
Mito_Roundness
Skel_EndPointsCount
Skel_BranchPointsCount
Skel_BranchPointsEndPointsRatio
Skel_Length
Skel_Width
MitoCluster_Area
MitoCluster_Perimeter
MitoCluster_Elongation
Mito_Elongation
Mito_Length
Cell_Area
Cell_Perimeter
Cell_Compaction
MitoCluster_Compaction
Mito_Compaction
DistToCellMembrane
DistToNuclei
RatioDistMemb0Nucl1
MitoCluster_EulerNumber
MitoCluster_Solidity
MitoCluster_Fractal8
MitoCluster_Fractal32
MitoCluster_Fractal64
MitoCluster_MeanIntensity
MitoCluster_MaxIntensity
MitoCluster_Count
Cell_MaxIntensity
+
-
FUSION
M2
RAMIFICATION
M3
COMPACTION
M4

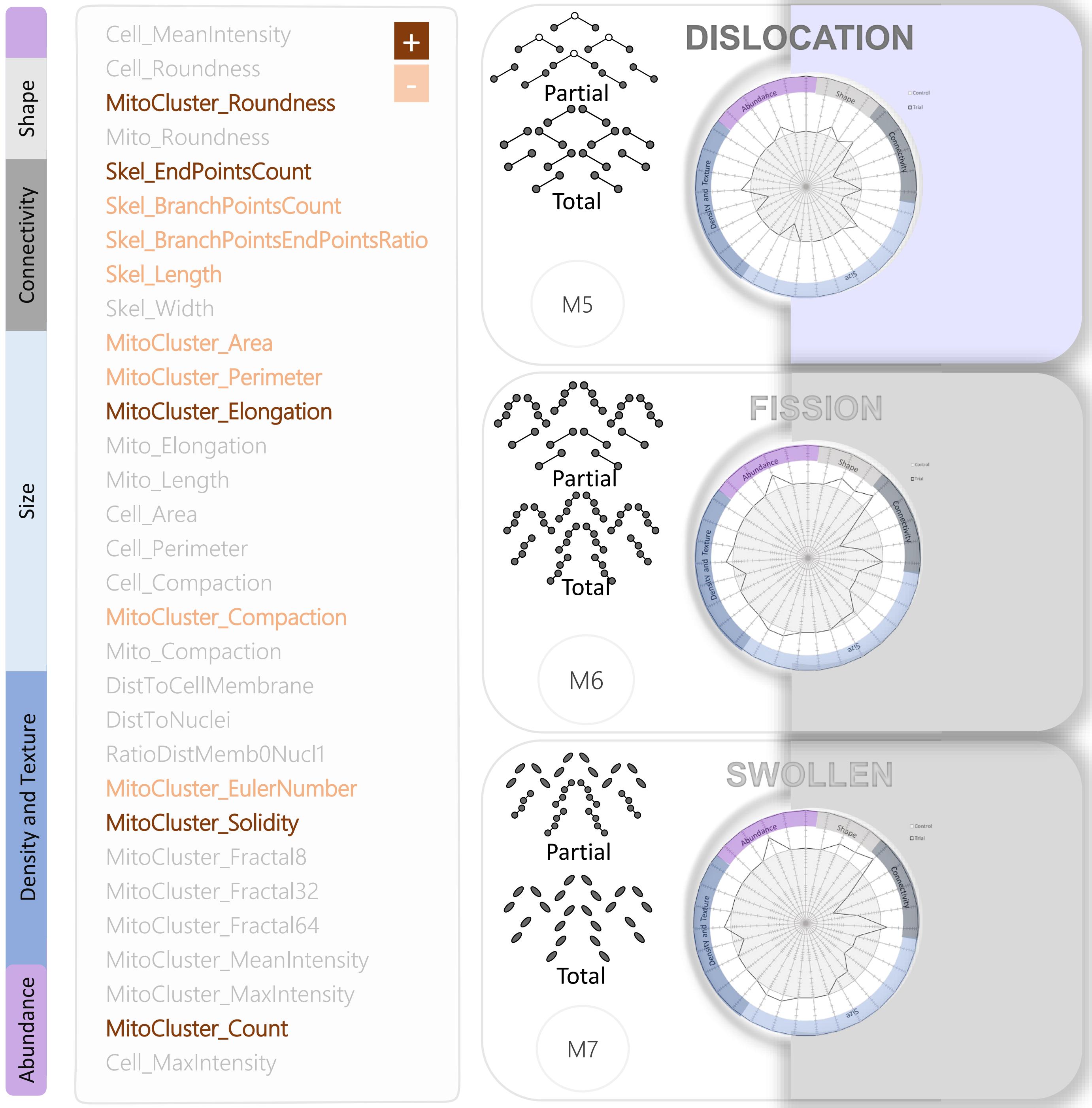
Shape
Connectivity
Size
Density and Texture
Abundance
Cell_MeanIntensity
Cell_Roundness
MitoCluster_Roundness
Mito_Roundness
Skel_EndPointsCount
Skel_BranchPointsCount
Skel_BranchPointsEndPointsRatio
Skel_Length
Skel_Width
MitoCluster_Area
MitoCluster_Perimeter
MitoCluster_Elongation
Mito_Elongation
Mito_Length
Cell_Area
Cell_Perimeter
Cell_Compaction
MitoCluster_Compaction
Mito_Compaction
DistToCellMembrane
DistToNuclei
RatioDistMemb0Nucl1
MitoCluster_EulerNumber
MitoCluster_Solidity
MitoCluster_Fractal8
MitoCluster_Fractal32
MitoCluster_Fractal64
MitoCluster_MeanIntensity
MitoCluster_MaxIntensity
MitoCluster_Count
Cell_MaxIntensity
+
-
DISLOCATION
Partial
Total
M5
FISSION
Partial
Total
M6
SWOLLEN
Partial
Total
M7

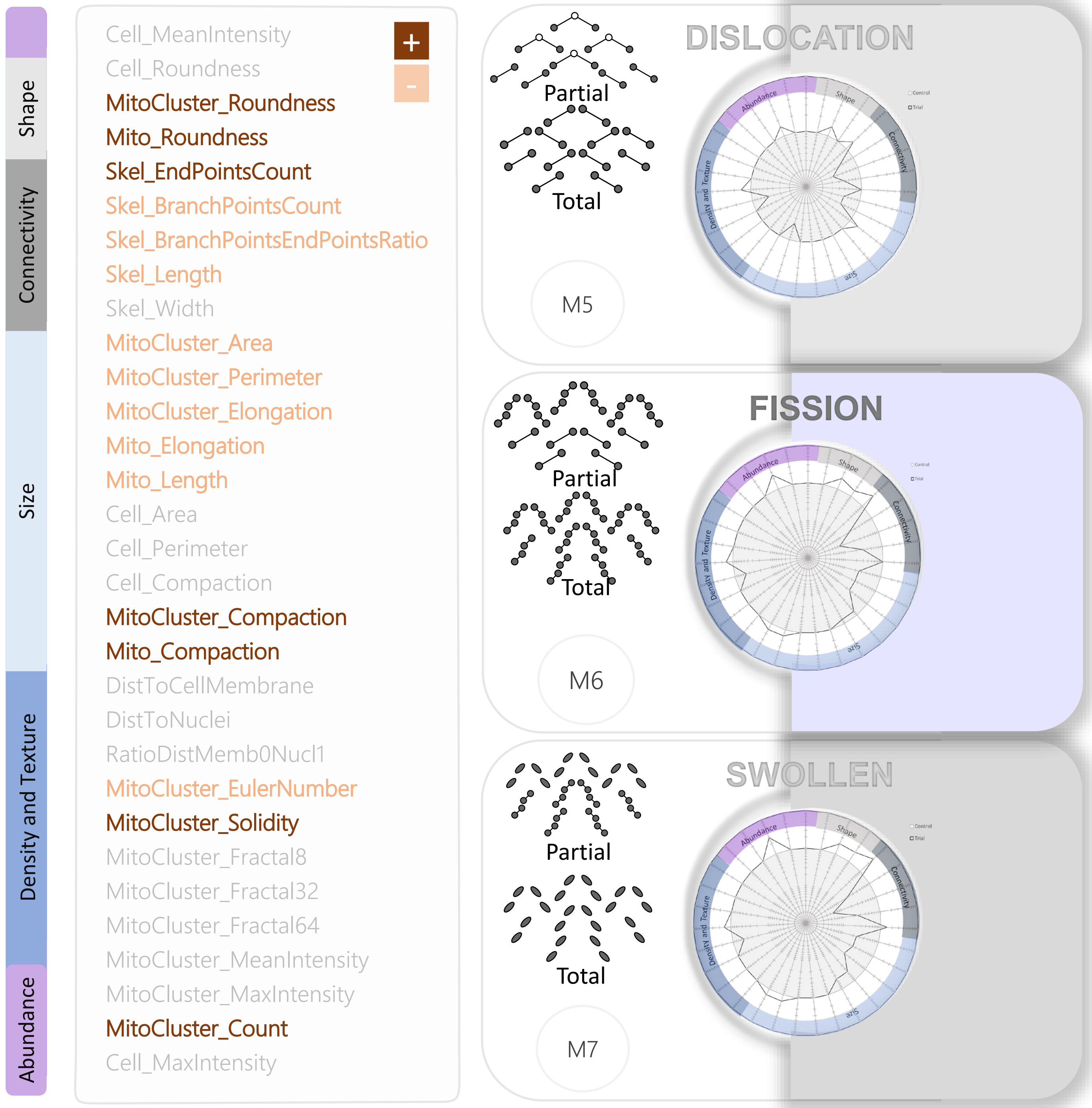
Shape
Connectivity
Size
Density and Texture
Abundance
+
-
Cell_MeanIntensity
Cell_Roundness
MitoCluster_Roundness
Mito_Roundness
Skel_EndPointsCount
Skel_BranchPointsCount
Skel_BranchPointsEndPointsRatio
Skel_Length
Skel_Width
MitoCluster_Area
MitoCluster_Perimeter
MitoCluster_Elongation
Mito_Elongation
Mito_Length
Cell_Area
Cell_Perimeter
Cell_Compaction
MitoCluster_Compaction
Mito_Compaction
DistToCellMembrane
DistToNuclei
RatioDistMemb0Nucl1
MitoCluster_EulerNumber
MitoCluster_Solidity
MitoCluster_Fractal8
MitoCluster_Fractal32
MitoCluster_Fractal64
MitoCluster_MeanIntensity
MitoCluster_MaxIntensity
MitoCluster_Count
Cell_MaxIntensity
DISLOCATION
Partial
Total
M5
FISSION
Partial
Total
M6
SWOLLEN
Partial
Total
M7

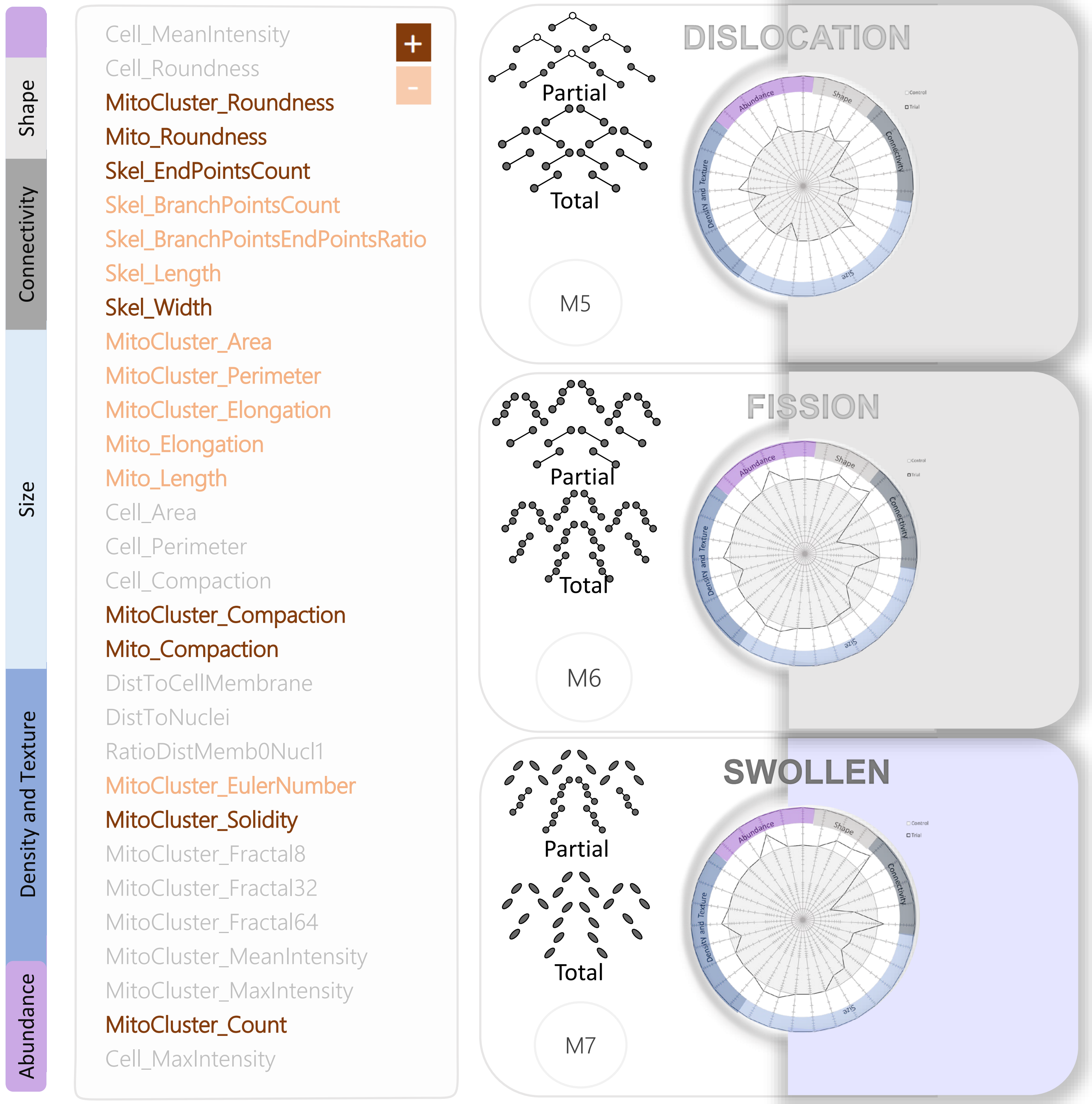

Shape
Connectivity
Size
Density and Texture
Abundance
Cell_MeanIntensity
Cell_Roundness
MitoCluster_Roundness
Mito_Roundness
Skel_EndPointsCount
Skel_BranchPointsCount
Skel_BranchPointsEndPointsRatio
Skel_Length
Skel_Width
MitoCluster_Area
MitoCluster_Perimeter
MitoCluster_Elongation
Mito_Elongation
Mito_Length
Cell_Area
Cell_Perimeter
Cell_Compaction
MitoCluster_Compaction
Mito_Compaction
DistToCellMembrane
DistToNuclei
RatioDistMemb0Nucl1
MitoCluster_EulerNumber
MitoCluster_Solidity
MitoCluster_Fractal8
MitoCluster_Fractal32
MitoCluster_Fractal64
MitoCluster_MeanIntensity
MitoCluster_MaxIntensity
MitoCluster_Count
Cell_MaxIntensity
+
-
DISLOCATION
Partial
Total
M5
FISSION
Partial
Total
M6
SWOLLEN
Partial
Total
M7
