# Supplementary material for: Quantitative imaging and semiotic phenotyping of mitochondrial network morphology in live human cells
Source: PLoS One. 2024 Mar 28;19(3):e0301372. doi: 10.1371/journal.pone.0301372 (PMC10977735; doi:10.1371/journal.pone.0301372)

S3 File. MitoSpider-Board and MitoSigil-Board.

All possible theoretical MitoSpider plots are represented. All schematic representations in the form of a MitoSigil, each corresponding to a theoretical Spiderplot, have been aligned on these sheets.

A

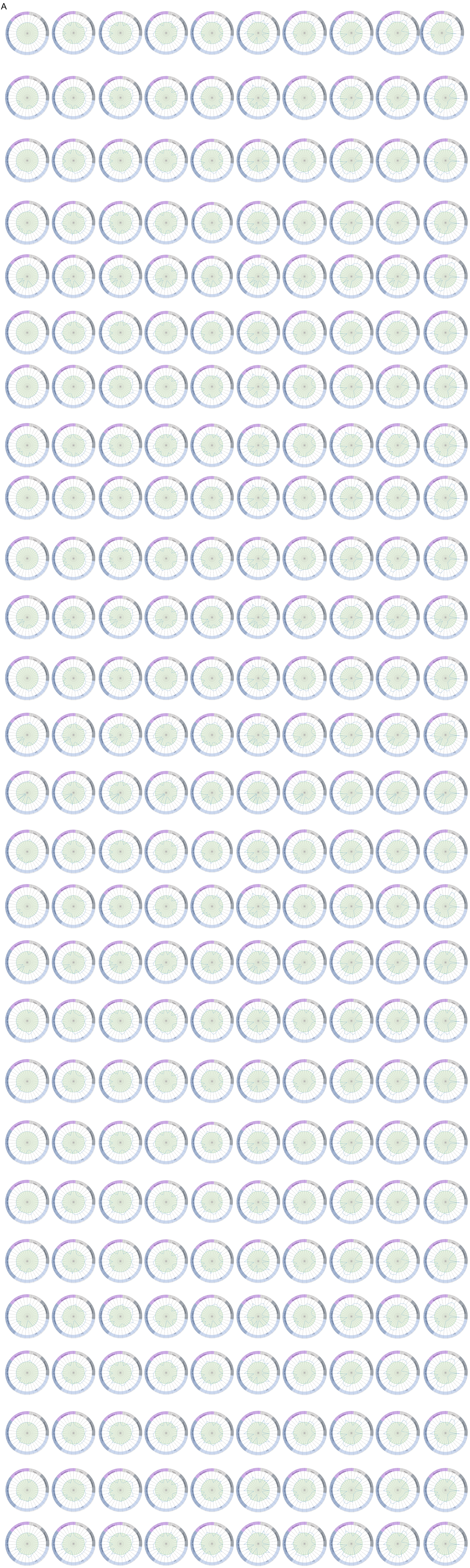

B

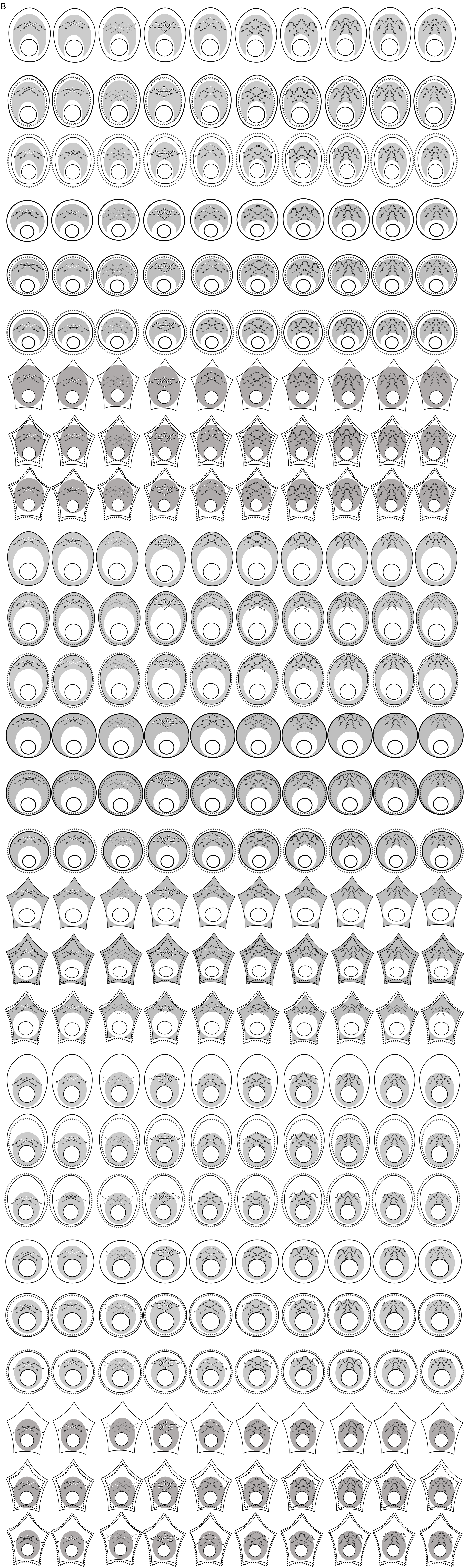

Supplement: S3 File — All possible theoretical MitoSpider plots are represented. All schematic representations in the form of a MitoSigil, each corresponding to a theoretical Spiderplot, have been aligned on these sheets. (PDF) [file pone.0301372.s003.pdf]
