# Supplementary figures and images for: Quantitative imaging and semiotic phenotyping of mitochondrial network morphology in live human cells
Source: PLoS One. 2024 Mar 28;19(3):e0301372. doi: 10.1371/journal.pone.0301372 (PMC10977735; doi:10.1371/journal.pone.0301372)

A

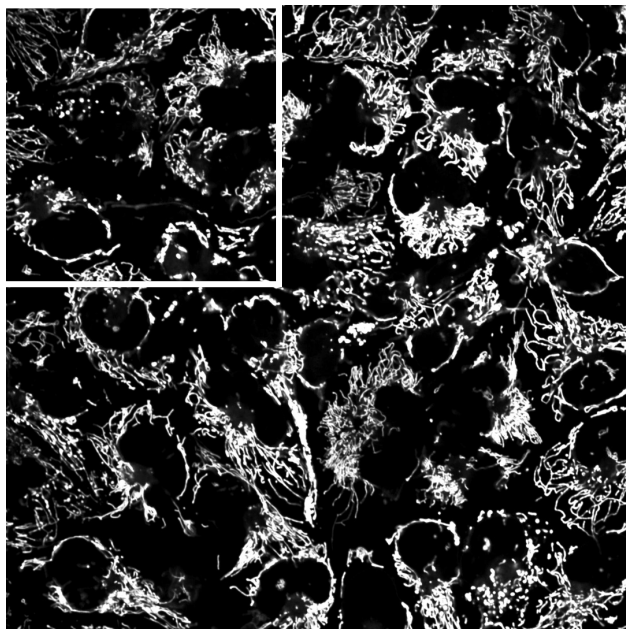

B

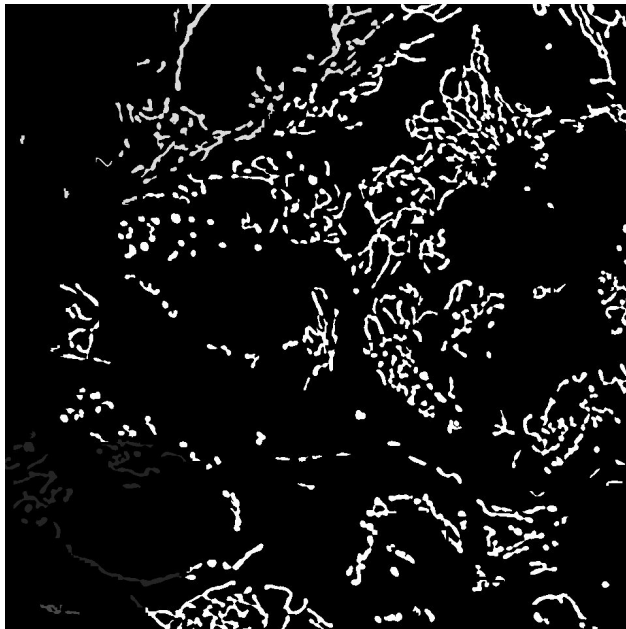

C

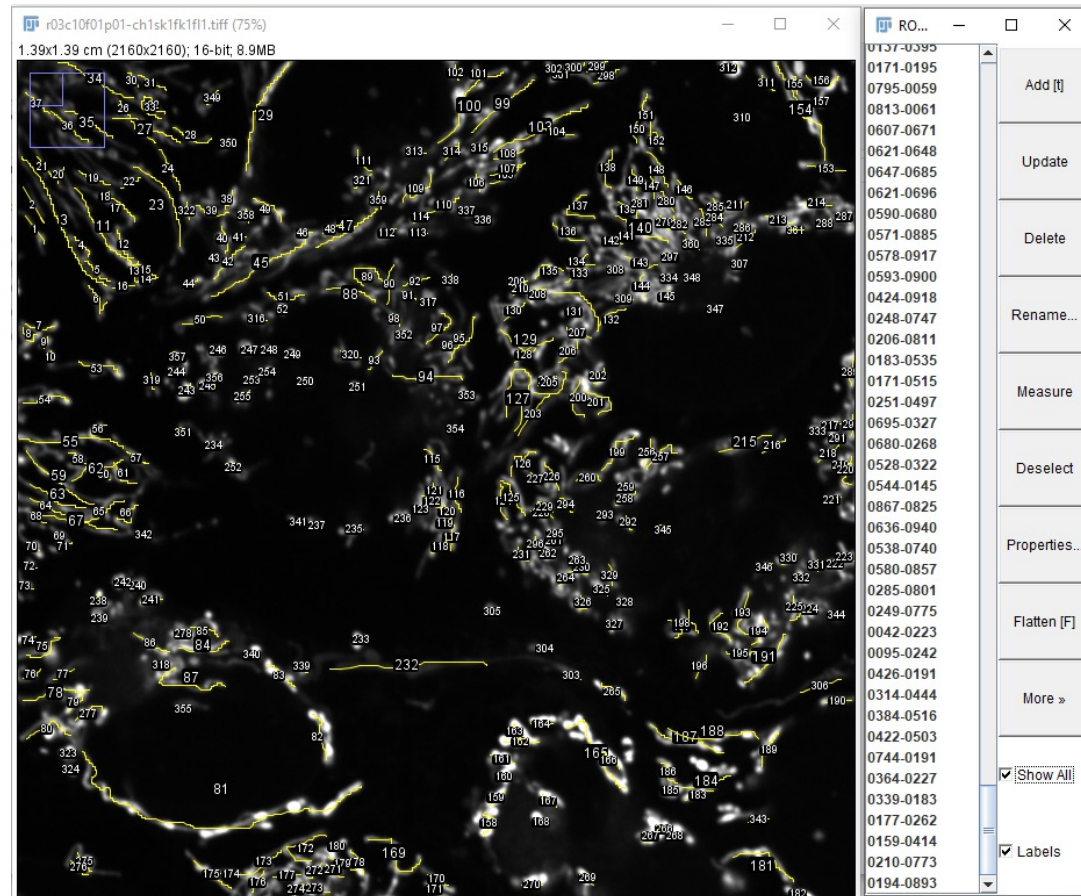

D

| Results |          |          |         |          |        |
|---------|----------|----------|---------|----------|--------|
|         | Area     | Mean     | Min     | Max      | Length |
| 1       | 1.941    | 491.072  | 84      | 10982    | 0      |
| 2       | 1.748E-5 | 820.129  | 363.141 | 1530.691 | 0.027  |
| 3       | 2.288E-5 | 234.655  | 112.043 | 420.796  | 0.035  |
| 4       | 8.446E-5 | 424.731  | 146.181 | 1168.000 | 0.119  |
| 5       | 1.456E-5 | 744.244  | 458.180 | 1122.000 | 0.020  |
| 6       | 2.122E-5 | 756.793  | 490.000 | 1105.000 | 0.027  |
| 7       | 2.247E-5 | 535.283  | 162.514 | 1013.880 | 0.033  |
| 8       | 1.623E-5 | 1050.663 | 618.610 | 1847.750 | 0.024  |
| 9       | 1.415E-5 | 1049.910 | 327.886 | 1723.440 | 0.020  |

E

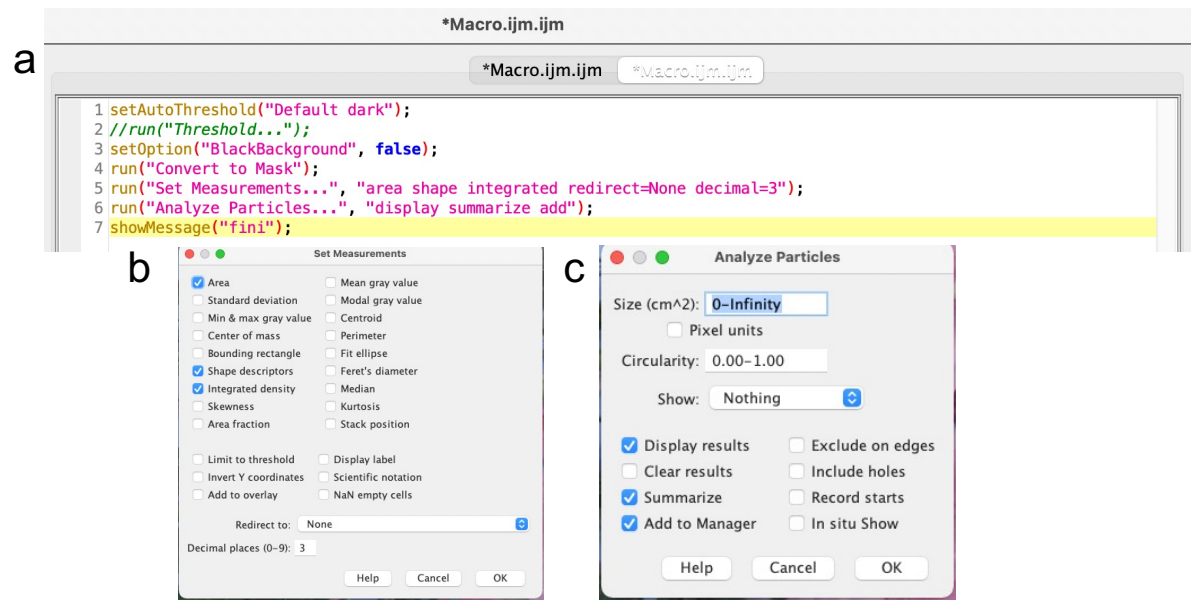

F

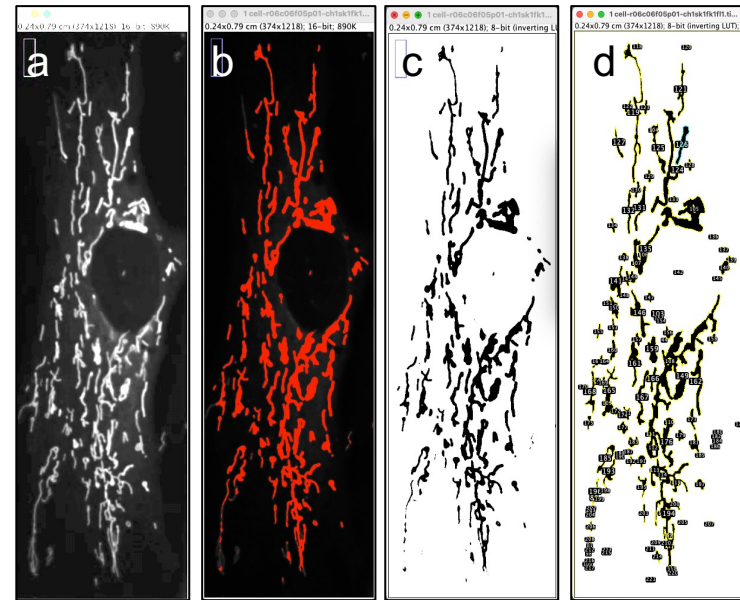

G

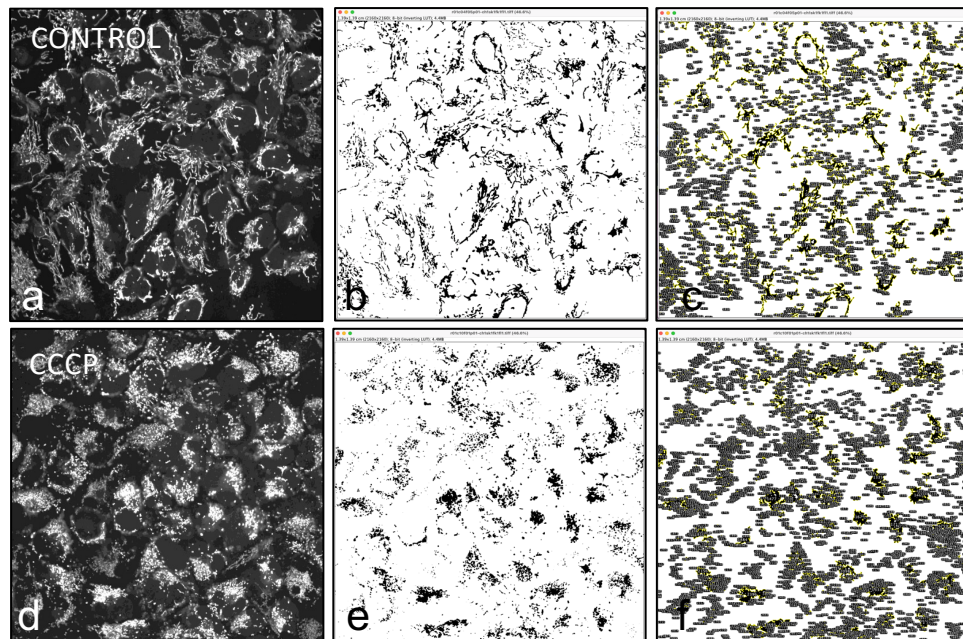

H

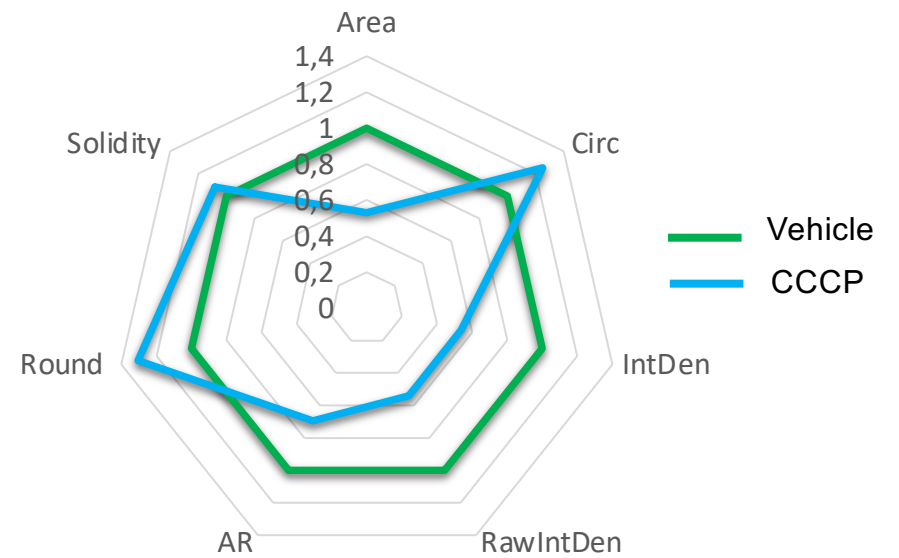

I

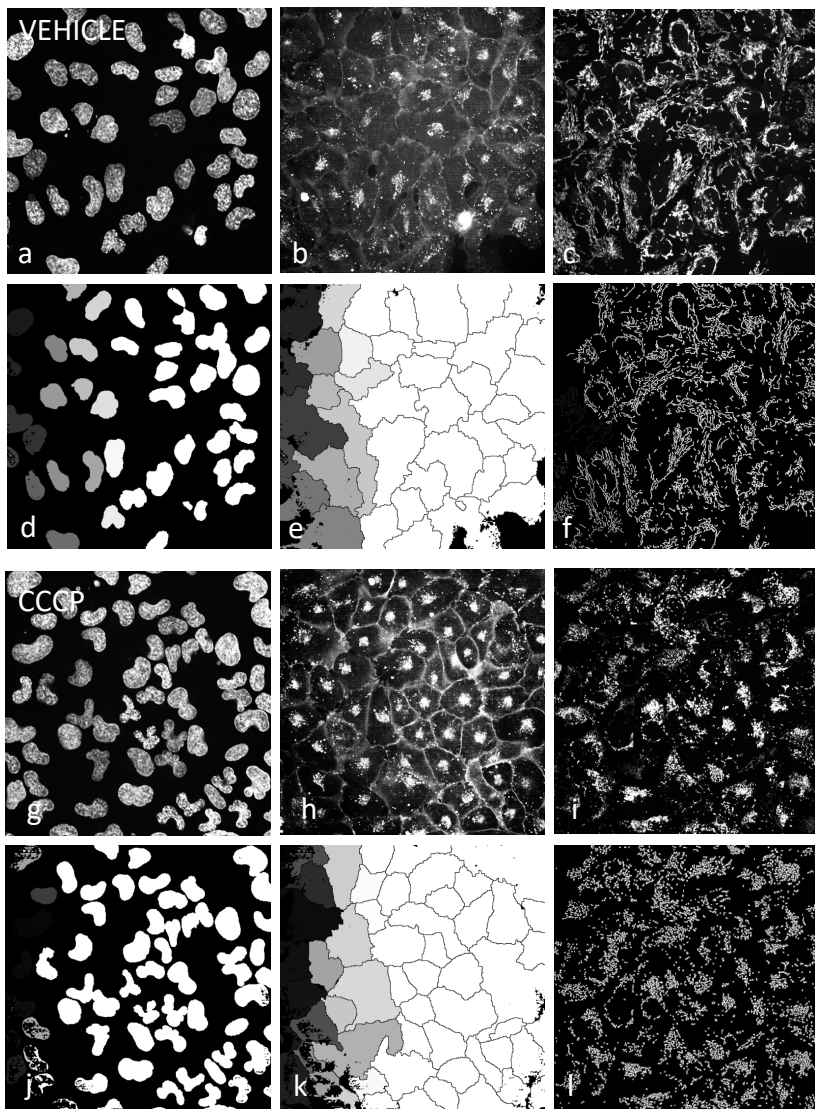

J

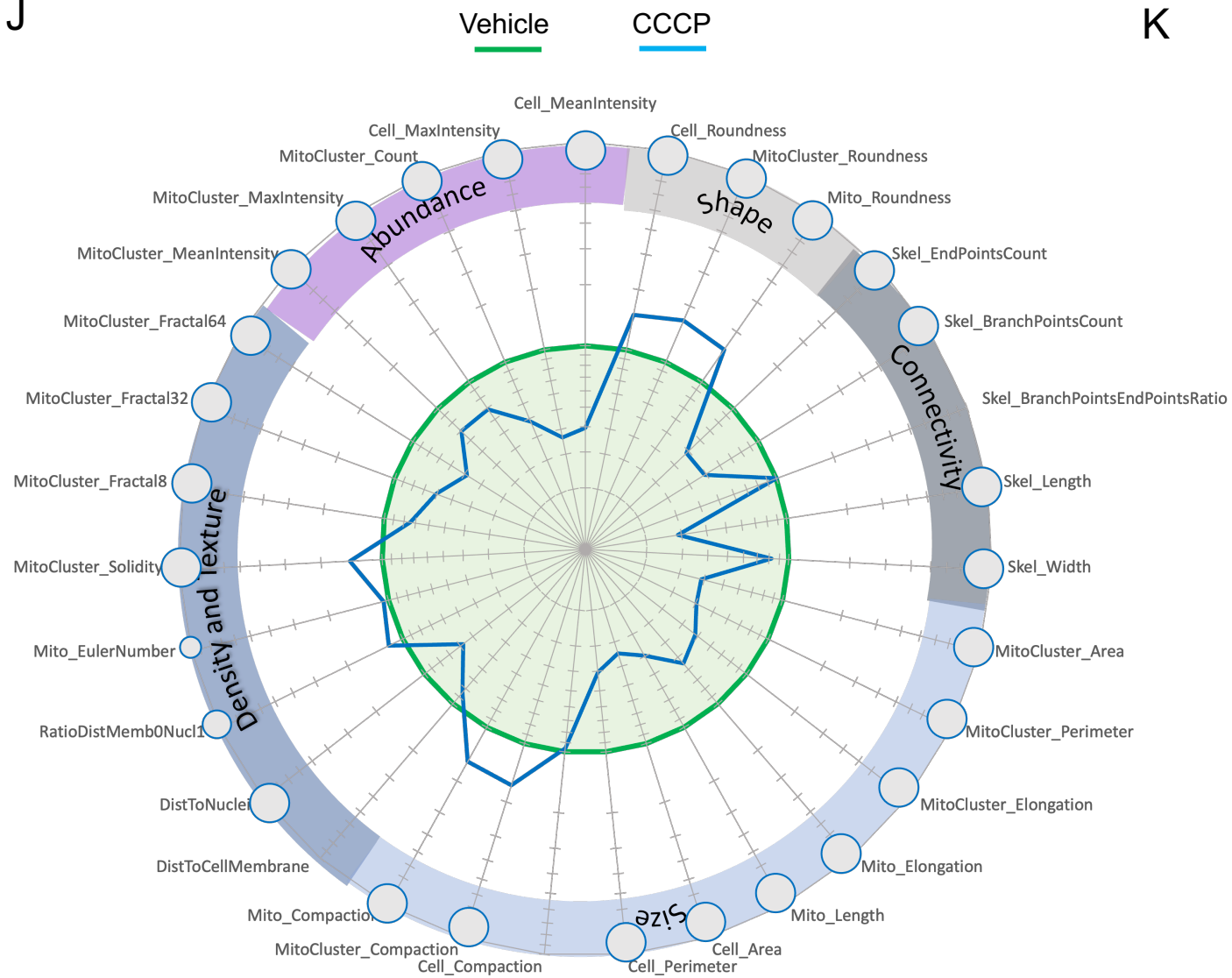

K

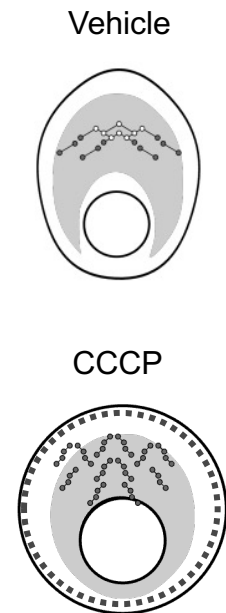

Supplement: S4 File — Human osteosarcoma U2OS cells were treated with vehicle (0,5% DMSO) or 20μM CCCP for 2h 30 min before staining with with 2.5μg/mL Hoechst 33342, 5 μg/mL Cell Mask Green and 250nM Mitotracker Deep Red and confocal imaging. Manual analysis: A-D; Semi-automatic analysis: E-H; MitoTouch analysis: I-K. (A) The framed area (956x955 pixels) equivalent to 19.6% of the original image was analyzed (corresponding to a total of 6 cells). (B) MitoTouch’s segmentation in the same area for visual comparison. (C) Manual delineation of mitochondria (yellow segments) in 6 different cells. A total of 362 lines (FreeHand lines in ImageJ) were defined manually in 720s. Automatic quantification in this zone detected 417 mitochondria in 16s (time saving factor x45). Note that mitochondrial clusters cannot be delineated. (D) Quantification of five parameters (in contrast to the 31 parameters computed by MitoTouch): Area, Length, Intensity Mean, Min, Max using ImageJ’s mesure function. (E) Macro description steps and analyzed features (a). Specification of recorded measurements: Area, Shape descriptors (Circularity, Aspect Ratio, Roundness, Solidity) & Integrated Density (b). Settings adjustments for minimum size and maximum pixel area size and roundness value (c). (F) Original confocal image of a single cell (a). Threshold adjustment with dark background and red particles (b). Mask conversion: output is a binary image black and white, with foreground 255 and background 0, using an inverted LUT (c). Measurements and particle analysis (d). (G) U2OS cells treated with vehicle (0,5% DMSO) (a-c) or 20μM CCCP (d-f). Pre-processing step (mitochondria mask: b, e). Quantification step (c, f). (H) MitoSpider plot representation. Seven parameters were computed (in contrast to the 31 parameters offered by MitoTouch): Area, Circularity, Integrated Density (IntDen: Area multiplied by the Mean gray value), Raw Integrated Density (RawIntDen: the sum of all pixel values in the region of interest), Aspect R [file pone.0301372.s004.pdf]
